# Performance, physiology, and determinants of success in IRONMAN^®^ 70.3: A systematic review

**DOI:** 10.1371/journal.pone.0352506

**Published:** 2026-07-17

**Authors:** Isabel von Känel-Cordoba, Matthias Wilhelm, Luciano Bernardes Leite, Marilia S. Andrade, Pantelis T. Nikolaidis, Pedro Forte, Daniela Chlibkova, Sasa Duric, Thomas Rosemann, Katja Weiss, Beat Knechtle

**Affiliations:** 1 Faculty of Medicine, University of Bern, Bern, Switzerland; 2 Centre for Rehabilitation & Sports Medicine, Bern University Hospital, University of Bern, Bern, Switzerland; 3 Department of Physical Education, Federal University of Viçosa, Vicosa, Brazil; 4 Department of Physiology, Federal University of Sao Paulo, Sao Paulo, Brazil; 5 School of Health and Caring Sciences, University of West Attica, Athens, Greece; 6 Department of Sports, Higher Institute of Educational Sciences of the Douro, Penafiel, Portugal; 7 Department of Sports Sciences, Instituto Politécnico de Bragança, Bragança, Portugal; 8 Research Center for Active Living and Wellbeing (Livewell), Instituto Politécnico de Bragança, Bragança, Portugal; 9 Centre of Sports Activities, Brno University of Technology, Brno, Czech Republic; 10 Liberal Arts Department, American University of the Middle East, Egaila, Kuwait; 11 Institute of Primary Care, University of Zurich, Zurich, Switzerland; 12 Medbase St. Gallen Am Vadianplatz, St. Gallen, Switzerland; University of Urbino: Universita degli Studi di Urbino Carlo Bo, ITALY

## Abstract

**Background:**

IRONMAN® 70.3 events represent a rapidly expanding endurance discipline characterized by distinct physiological demands, performance determinants, and race-specific risk profiles. Despite their global popularity, no systematic review has synthesized evidence exclusively focused on this distance.

**Objective:**

To consolidate current knowledge on performance predictors, physiological responses, training characteristics, nutritional strategies, environmental influences, and medical considerations in IRONMAN® 70.3 triathletes, and to identify gaps requiring further investigation.

**Methods:**

A systematic search of PubMed, Scopus, SciELO, EBSCO and Google Scholar was conducted up to 25^th^ November 2025. Search terms were developed according to PRISMA guidelines and included variations of ‘Ironman 70.3’, ‘half triathlon’ and ‘middle-distance triathlon’. Eligible studies reporting physiological, anthropometric, nutritional, environmental, medical, or performance-related outcomes specific to IRONMAN® 70.3. Risk of bias was assessed using the Newcastle–Ottawa Scale, the Cochrane RoB tool, and the NIH Quality Assessment Tool, according to study design.

**Results:**

A total of 86 studies were included, predominantly observational in design, with sample sizes ranging from 1 to 852,721 participants, mostly trained male triathletes aged 25–39 years. Participation has increased across age groups, with pronounced growth among female and masters triathletes. Peak performance in professional male triathletes is reached at approximately age 28, and in female triathletes at age 32. Across large datasets, cycling appeared to be the strongest predictor of overall race time, accounting for the largest proportion of performance variance. Physiologically, competition was associated with transient reductions in immune function, reversible muscle damage, and shifts in hydration and electrolyte balance, while higher intracellular water and efficient fat oxidation were associated with better outcomes.

**Conclusions:**

Evidence specific to IRONMAN® 70.3 is limited by small sample sizes, heterogeneous designs, male-dominated cohorts, and insufficient sex-specific analyses. Future research should distinguish recreational from elite triathletes, incorporate balanced sex representation, and apply standardized physiological and environmental monitoring to refine targeted recommendations for performance, health, and safety.

## Introduction

IRONMAN® 70.3, commonly known as the Half IRONMAN®, consists of a 1.9 km swim, 90 km cycling and 21.1 km run, placing it between Olympic-distance and full-distance IRONMAN® triathlon [1,2]. Participation has increased markedly across age groups and both sexes in recent years, reflecting broader growth in endurance sports and event accessibility [3–9].

Triathlon performance depends on the integrated demands of swimming, cycling and running, influenced by discipline-specific physiological, biomechanical, environmental, and metabolic factors [1,10]. Previous research has examined these determinants across different triathlon distances [6,11–14]. However, findings are often based on small sample sizes, heterogeneous study designs and mixed race formats, limiting their generalizability and direct applicability to IRONMAN 70.3®.

Although the increasing number of participants suggests a greater need for specific information on IRONMAN® 70.3, research on this topic remains fragmented, with small, predominantly male samples, heterogeneous methodologies and inconsistently reported environmental and physiological metrics. No prior synthesis has systematically integrated evidence across performance determinants, physiological adaptations, environmental modifiers, nutritional strategies and medical aspects for this distance. The present review therefore consolidates multidisciplinary findings to support triathletes, coaches, clinicians and race organizers, and identifies key gaps, including sex-specific responses, age-related differences and mechanistic pathways that require targeted investigation.

## Methodology

### Search databases and terms

This systematic review was conducted in accordance with PRISMA 2020 recommendations, with all methodological steps documented prospectively following an initial scoping phase. The PRISMA 2020 checklist is provided in S1 File. Registration in PROSPERO was not possible because retrospective submission is not permitted. A comprehensive search was performed in PubMed, Scopus, SciELO, EBSCO and Google Scholar, selected for their relevance to sports and health sciences research [15,16]. The search covered all records up to 25 November 2025, without language restrictions. Search terms were developed using free-text principles [17] and included variations of: ((Ironman AND Half Triathlon) OR (Ironman AND 70.3) OR (Ironman AND Half AND Triathlon) OR (middle-distance Triathlon)). Google Scholar results were screened through the first seven pages, with entries on pages 6 and 7 excluded due to irrelevance. Three additional studies were identified via reference and citation tracking.

### Inclusion and exclusion criteria

Studies were eligible if they investigated the IRONMAN® 70.3 distance, involved human participants, and reported physiological, anthropometric, nutritional, environmental, medical or performance-related outcomes. Observational (cross-sectional, longitudinal, retrospective), experimental and interventional designs were included. Studies analysing other triathlon distances were eligible only when IRONMAN® 70.3-specific outcomes were clearly distinguishable. Exclusion criteria encompassed animal or in vitro studies, multi-stage or ultra-endurance events, ultra-swimming, ultra-cycling, mountain biking, cross-country skiing and studies lacking extractable 70.3-specific data.

### Study selection process

After removal of duplicates, 86 studies met the inclusion criteria. Title and abstract screening were performed independently by three reviewers IvK, BK and KW, followed by full-text assessment when eligibility remained unclear. Any discrepancies were resolved through discussion until consensus was reached.

### Data extraction and categorization

The dataset is provided in S2 File. For each included study, data were extracted on participant characteristics, sample size, race conditions, environmental context, performance metrics, training variables, anthropometric data, nutritional strategies, physiological and biochemical markers, immune and inflammatory responses, and medical outcomes. Extracted variables were categorized into thematic domains to support structured synthesis and cross-study comparison.

### Risk of bias assessment

Given the heterogeneity of study designs, risk of bias was assessed using tools appropriate to each methodology: the Newcastle–Ottawa Scale for observational studies, the Cochrane Risk of Bias Tool for interventional trials, and the NIH Quality Assessment Tool for cross-sectional and physiological research. Major methodological challenges included small samples, male-dominated cohorts, inconsistent measurement timing, variable laboratory versus field conditions and incomplete reporting of environmental characteristics.

### Methodological limitations and future research priorities

Comparative analyzes with other races such as the full distance IRONMAN® and the Olympic distance triathlon were used to determine IRONMAN® 70.3 specificities and general findings. Data heterogeneity, stemming from non-standardized performance metrics, scarce interventional designs, and variable environmental conditions, impeded comparability. Small sample sizes in studies on physiological adaptation, training programs, and nutritional strategies further constrained interpretability. Generalizability is additionally limited by a pronounced male bias due to participation patterns and the rarity of female-specific cohorts. Additionally, the prevalence of cross-sectional designs and inadequate control of environmental factors limited causal inferences.

## Results

**Fig 1:**
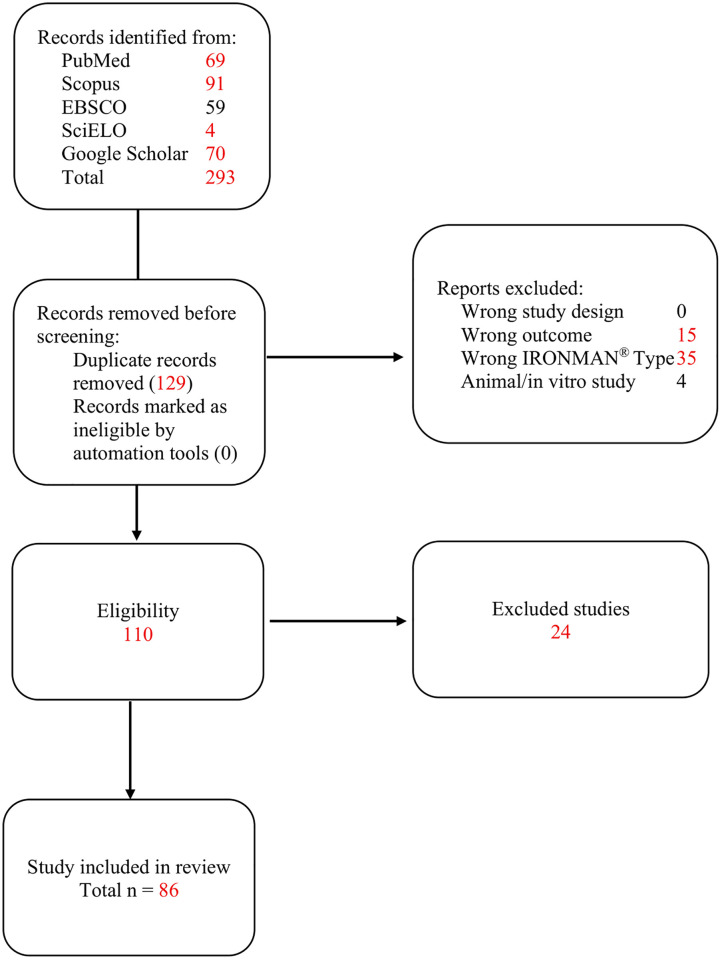
PRISMA flow diagram of study selection for IRONMAN^®^ 70.3. The Flow diagram illustrating identification, screening, eligibility, and inclusion of studies found for IRONMAN^®^ 70.3. Based on the study by Siddaway et al. [15].

Following the screening process, a total of 86 studies were included in this review (Fig 1). The included studies comprised case studies, observational studies, and experimental studies and primarily focused on trained, male, and young triathletes, although the study populations varied in terms of sample size and gender distribution across the different outcome categories. The results are organized in descending order by number of studies found into physiological adaptations, competition-related complications and medical strategies, performance determinants and competition characteristics, nutritional strategies, age and gender in peak performance, as well as training characteristics and performance predictors.

### Physiological adaptations

Twenty-two studies examined physiological responses to IRONMAN® 70.3, predominantly in small cohorts of trained male triathletes aged 25–39 years [18–36] (Tables 1 and 2). Nineteen studies investigated acute post-race changes, while three captured both acute and chronic adaptations [31,33,35]. Most measurements were conducted immediately before and after the race, with delayed follow-up assessments performed under laboratory conditions.

**Table 1:** Physiological adaptation.

Study participants	Title, Authors, Year	Result	Reference
n = 7 M	Changes in Kidney Functions during Middle-distance Triathlon in Male Athletes Poortmans et al. 2015	• Albuminuria increased 30-fold after the swimming portion no change was observed during running and cycling. • The glomerular filtration rate excretion, the β_2_-microglobulin and the retinol-binding protein remains constant throughout the triathlon. • Glomerular membrane permeability was 13 times increased during the swimming event • The reabsorption capacity was impaired after each unit.	[18]
n = 37 M	Anthropometric Characteristics of Chilean Amateur Triathletes: A Pilot Study Sanhueza et al. 2017	• Age differences in skin mass between triathletes aged ≤24 years and ≥35 years, with these two groups having a lower percentage of fat mass and greater muscle mass. • The morphological characteristics of amateurs differ from those of professional triathletes, with endomorphism higher in amateurs aged ≥35 years and ectomorphy higher in amateurs aged ≤24 years. • The body composition of the amateur triathletes had high fat mass (23.9%), moderate muscle mass (48.0%) and low values for bone mass (11.3%), residual mass (11.6%) and skin mass (5.2%).	[19]
n = 6 M	Chlorine behavior in a half-Ironman triathlon. Bürger-Mendonça et al. 2007	• After a race, chloride levels dropped by 5.68% compared to before the race, regardless of the duration of the race.	[20]
n = 10 M	MAGNESIUM-ION BEHAVIOR IN A HALF-IRONMAN TRIATHLON Bürger-Mendonça et al. 2008	• Compared to triathletes who did not participate in the race, the magnesium concentration decreased by 35.23%.	[21]
n = 6 M	SIGNIFICANT REDUCTION IN SODIUM PLASMA AFTER THE SEMI-IRONMAN TRIATHLON IN BRAZILIAN TRIATHLETES. Bürger-Mendonça et al. 2009	• After a race, the sodium plasma concentration decreased by 3.38 mmol/L compared to before the race, regardless of the duration. • The reduction was still within the normal range.	[22]
n = 26 M	Changes in serum free amino acids and muscle fatigue during a half-Ironman triathlon Areces et al. 2015	• The concentration of essential amino acids decreased by −27.1%, valine, leucine, and isoleucine decreased by −32.8%, the ratio of tryptophan to valine, leucine, and isoleucine increased by 42.7%, and the ratio of non-essential amino acids increased by −24.4% after the race. • No correlation was found between these changes and muscle fatigue variables or post-race creatine kinase concentration.	[23]
n = 13 M, 3 F	Oxidative Stress in Half and Full Ironman Triathletes Knez et al. 2007	• After a race, plasma MDA increases by 25.1%, while GPX, SOD, and CAT decrease, leading to increased oxidative stress. • Triathletes have increased GPX (glutathione peroxidase) activity at rest, indicating increased antioxidant capacity. • Supplementation with vitamins E and C does not appear to reduce oxidative stress.	[24]
n = 12 M	Half-Ironman leads to changes in the kidney function of triathletes Puggina et al. 2014	• After the half triathlon, urinary protein excretion increased by about 1000%, creatinine excretion by 100.72% and 78.1%, respectively, and red blood cell count by about 5,000%.	[25]
n = 13 N/A	Acute effects of triathlon races on biomarkers of oxidative stress Mrakic-Sposta et al. 2020	• ROS production increases by 20% after a race, PC production by 101%, TBARS by 57%, neopterin by 19% and aminothiols by 28%. • IL-6 concentration is inversely correlated with exercise activity. • Triathletes with more years of experience show increased oxidative stress formation and inflammatory response.	[26]
n = 19M, 4 F	ACTN3 X allele carriers had higher levels of muscle damage during a half-Ironman Del Coso et al. 2016	• RX and XX alleles in the ACTN3 R577X polymorphism carriers showed higher serum creatine kinase concentrations (682 ± 144 vs. 472 ± 269 U/L) and self-reported more severe muscle pain of the legs (7.7 vs. 6.3 cm from 1–10 cm). • The race performance showed no significant change.	[27]
n = 23 M	Death of neutrophils due to a triathlon competition among elite athletes Levada-Pires et al. 2008	• The 12 professional triathletes showed a 3-fold increase in DNA fragmentation in neutrophils, a 2.5-fold increase in ROS production, and a 2-fold increase in externalization of phosphatidylserine external positioning. This indicates apoptosis of neutrophils.	[28]
n = 57 N/A, HIR 24 N/A	Induction of lymphocyte death through short- and long-term triathlon competitions Levada-Pires et al. 2009	• Experienced triathletes showed a 22% increase in lymphocytes with loss of membrane integrity and a 1.6-fold increase in ROS production after a race. The study therefore assumes necrosis of the lymphocytes.	[29]
n = 6 M	Liver overload in Brazilian triathletes after a half-Ironman competition is related to muscle fatigue Bürger-Mendonça et al. 2008	• ALT did not change significantly by +5.43 U/I, AST by +19.1 U/I and ALP by +42.17 U/L changed significantly but remained below the pathological limit. • There is no correlation to muscle or liver damage, nor is there a correlation to the end times.	[30]
n = 42 M	Changes in water balance related to the level of performance in the Half Ironman Lenka et al. 2015	• Professional triathletes show a higher total body water of 63.73% to 60.97% of body weight before a race and a higher intracellular water of 2.17%, about 5.5% more than the amateurs. • After a race, the professional triathletes and amateur triathletes showed no differences in the change in the water balance. • In both groups, there was significant weight loss and water loss, which did not correlate with the end time. Total Body water loss was in elite 0.98l and in recreational triathletes 0.75l.	[31]
n = 11 M	Effect of ultra-resistance exercises on oxidative stress parameters Dornelles Schneider et al. 2009	• After the race, there was a significant increase in plasma uric acid concentrations of 48% and a significant decrease in the activity of the superoxide dismutase enzyme by 10%, indicating uric acid concentrations in the plasma. • The lipid damage assessed using TBARS and chemiluminescence techniques and the protein damage assessed using the carbonyl technique show no statistically significant difference. • The enzymes catalase, glutathione peroxidase and phenolic compounds proved to be stable.	[32]
n = 12 M	Changes in body composition after endurance training and triathlon competitions Puggina et al. 2011	• The triathletes were measured before the start of training, after 12 weeks of training with 20% interval and 80% duration and 30 minutes after the race. The body mass 71.83 to 74.22 to 72.15 kg shows no significant change, the proportion of body fat related to skin folds also remained stable at 10.98 to 10.92 to 10.40% and the urine density and pH remained almost unchanged. • The bioelectrical impedance measurement with 13.54 to 13.91 to 9.45% and the free fatty acids with 0.16 to 0.15 to 1.69 mEq/L showed different results after races. • The triathletes showed no dehydration after the race.	[33]
n = 34 N/A	Half Ironman influence on blood markers Gallo-Salazar et al. 2015	• The decrease in body mass from 72.8 ± 6.4 to 69.5 ± 6.4 kg indicates dehydration. • The increase in myoglobin from 32.8 ± 13 to 654.8 ± 451.3 μg• L-1 as well as the increase in CK from 169.3 ± 86.2 to 564.5 ± 428.9 U• L-1 and the increase in LDH from 318.4 ± 56.2 to 479.0 ± 78.6 U• L-1 indicate significant muscle damage. • The increase in calcium from 9.5 to 10.3 mmol• L-1 and of sodium from 140.6 ± 1.4 to 143.0 ± 2.0 mmol• L-1 and the increase in osmolality from 293.9 ± 7.3 to 301 ± 7.0 μg L-1 indicate a loss of electrolytes. Potassium and chlorine showed no significant changes.	[34]
n = 12 M	Effects of the training season and half an Ironman on muscle damage and inflammation indicators Puggina et al. 2016	• The change in blood values was measured before training, after 10 weeks of training and after the race. • A significant change after the race can be seen in the CK (UI/L) from 22.25 and 20.80 to 235.50 hrs, as well as in the LDH (UI/L) from 41.71 and 19.87 to 191.00 hrs. IL-6 values (pg/ml) also increased from 77.09 and 93.39 to 228.48 and IL-10 (pg/ml) from 88.49 and 89.56 to 193.31 and cortisol (μg/dl) from 14.60 and 23.96 to 37.47, respectively. • The CRP (mg/L) showed no significant change from 8.42 and 5.77 to 7.62, respectively.	[35]
n = 10	An ultra-endurance event results in changes in circulating regulatory T cells, CD4 + naïve, and CD8 + effector memory T cells in the 48 hours following the race Lithgow et al. 2024	• Neutrophils and monocytes increase significantly from 1 day after the race to before the race. The neutrophils of 2200 cells μL − 1–3249 μL − 1 and the monocytes of 422 cells μL − 1–1585 cells μL − 1, after 48 hours they are similar to the baseline value again. The lymphocytes remained at similar values. • The absolute cell count μL − 1 of CD3 + T cells and CD4 + T cells showed no significant changes. The proportion of CD8 + T cells to CD3 + T cells increased from 6.7% to 8.1% from 24h to 48h after the race. • There was a significant increase in regulatory T cells 24 hours after the race, which dropped almost to the original level 48 hours after the race.	[36]
n = 42 N/A	Endurance training shifts the balance between Th17 cells and regulatory T cells Perry et al. 2013	• Hyperreactivity of the respiratory tract and asthma have been documented in endurance sports. • A connection is suspected with the Th17 cells in the peripheral blood, which increased from 1.16% to 2.96%, and the declining peripheral regulatory T cells from 6.4% to 2.62%.	[37]
n = 27 M, HIR13 M, IR 14 M	Acute effects of different degrees of ultra-endurance training on systemic inflammatory responses Comassi et al. 2015	• Compared to IR, HIR showed a higher basal level of interleukin (IL)-6, tumor necrosis factor (TNF)-α, IL-1β, IL-10, than IR in the acute phase. • Irisin only rose in the HIR. • The white blood cells were directly related to the change in the monocyte chemoattractant protein 1.	[38]
Overview	Medical considerations in triathlon competition Dallam et al. 2012	• After the race, the triathletes showed reduced killer cells and proliferating lymphocytes and leukocytes, suggesting short-term immunosuppression. • Increased sympathetic fatigue is attributed to a greater reduction in plasma β endorphins, norepinephrine, and adrenocorticotropic hormone. • After the race, the triathletes showed hemolysis, which manifested itself in a reduced serum haptoglobin.	[39]
n = 22 M	*In vivo* cell-mediated immunity and vaccination response after prolonged, intensive training Bruunsgaard et al. 1997	• Vaccination with tetanus-diphtheritis-toxoid showed a reduced cutaneous response with hardening and erythrem. The pneumococcal polysaccharide vaccination showed no differences. No absolute figures could be derived from the study. • Antibody production 2 weeks after vaccination showed no differences between subjects in groups 22 vs. 33, suggesting an influence on non-specific acute immunity after exercise.	[40]

**Abbreviations**: F for women, M for men, ROAD for position in the stationary bike and TRI, for aerodynamic position in the stationary bike. IR means Ironman, HIR for Half-Ironman, Plasma MDA means malondialdehyde, GPX means glutathione peroxidase, SOD means superoxide dismutase, CAT means catalase. PC stands for protein carboxylic acids, CK for creatine kinase, LDH for lactate dehydrogenase, CRP for reactive C protein. TBARS stands for thiobarbituric acid-reactive substances.

**Table 2:** Injury prevention.

Study Participants	Title, Authors, Year	Result	Reference
n = 71 N/A, HIR 30 N/A	Swimming-induced pulmonary edema: pathophysiology and risk reduction with sildenafil Moon et al. 2016	• Increased MPAP and PAWP appear to be positively associated with SIPE. • In individuals with a history of this condition, a single dose of 50 mg of sildenafil taken orally may reduce this pressure.	[41]
n = 749 M, 662 F	Swimming-induced pulmonary edema in triathletes Miller et al. 2010	• 1.4% of the participants surveyed had pulmonary edema. • The likelihood of pulmonary edema while swimming was highest in patients with hypertension, which can be associated with diastolic dysfunction. Other risk factors are the distance (from a half triathlon), the female sex and fish oil consumption.	[42]
n = 9 M	Effects of the Half Ironman Competition on the Development of Late Potentials Warburton et al. 2000	• Two triathletes showed a single SAECG abnormality, i.e., H. No LP, before a half triathlon. The same triathletes showed two out of three SAECG abnormalities 2–3 hours after the race. Late potential after 24 hours remained for one of the two triathletes. • There is currently no evidence that these triathletes are at increased risk for SCD.	[43]
n = 14 M	Cardiac fatigue following prolonged endurance exercise of differing distances Whyte et al. 2000	• Total CK, CKMM, CKMB, and troponin-T were significantly elevated immediately after the race and also after 48 hours (P < 0.05), with the exception of troponin-T, which returned to resting levels in experienced triathletes. • After the race, there was reversible left ventricular diastolic and systolic depletion, as well as increased late diastolic filling and reduced early diastolic filling, suggestive of left ventricular diastolic stiffness.	[44]
n = 9 M	Altered cardiac function and minimal cardiac damage during prolonged exercise Shave et al. 2004	• Experienced triathletes showed a reduction in myocardial contractility after a race and a consequent change in diastolic filling pattern, but it returned to baseline levels within 24 hours. • A release of troponin-T was observed in 44% of the subjects. Due to the low elevation and the short duration of elevation, cytosolic degradation is suspected. • EICF and EICD after the race can have various causes, such as downregulation of cardiac β-adrenoceptors, accumulation of free fatty acids, myocardial anesthesia due to ischemia time, altered Ca2 + sequencing.	[45]
n = 36 M	Compression socks do not improve muscular performance during a half-Ironman triathlon Del Coso et al. 2014	Compression socks show no benefit in preventing muscle damage, improving results, or improving muscle function.	[46]
n = 777 N/A, HIR 54 N/A	Evaluation for ulnar neuropathy at the elbow in Ironman triathletes Bales et al. 2012	• Compared to pre-race and post-race, a positive Tinel sign was showed by 39.5% to 70.4% and 41.5% vs. 75.9% showed a positive flexion/compression test. • There were no significant differences between men (33.3%) and women (41.7%) P = 0.594. • The Aeorbar was suspected to be the cause.	[47]

Abbreviation: Swim-Induced Pulmonary Edema SIPE, Mean Pulmonary Arterial Pressure MPAP, Pulmonary Arterial Wedge Pressure PAWP, LP Mean Late Potential, SAECG Mean Signal-Averaged ECG, SCD Mean Sudden Cardiac Death, CK for Creatine Kinase, CKMM Mean Creatine Kinase-MM, CKMB Mean Creatine Kinase-MB, EICF Means Exercise-Induced Cardiac Fatigue, EICD Means Exercise-Induced Cardiac Injury.

Across eight studies, body composition, hydration and electrolyte responses were measured (male-only cohort, n = 6–42) [5,6,13–15,48–50]. Bioimpedance analyses demonstrated reductions in fat mass (e.g., from 13.91% to 9.45%) and increases in circulating free fatty acids (0.15 to 1.69 mEq/L) [33]. Professional triathletes exhibited higher total body water (63.7% vs. 61%) and intracellular water, compared with recreational athletes [31]. The fluid losses during the race were comparable, 0.98l for professional athletes and 0.75l for recreational athletes, without association with overall race time [31]. Amateur triathletes showed higher fat mass (23.9%) and lower muscle (48%) and lower bone mass (11.3%) compared to professionals [5,6]. Electrolyte responses were found under ad libitum intake conditions. It reported modest declines in key electrolytes including sodium declined by 3.38 mmol/L (141.72 to 138.37 mmol/L) [22]. Calcium increased by 0.8 mmol/L [34], whereas potassium and chloride showed no significant alterations [20–22,34]. No values fell below clinical thresholds, and none were associated with performance outcomes.

During IRONMAN® 70.3, measurable alterations were found across multiple physiological systems, including skeletal muscle, renal and hepatic markers, pulmonary function, and cardiac responses. In four studies (n = 12–34 male, 4 female) markers of muscle damage documented significant increases, in creatine kinase (3- to 10-fold), myoglobin (32.8 to 654.8 mg/L) and lactate dehydrogenase (318.4 to 479 U/L; 41.71 to 191 U/L), due to the absence of pathological threshold values.

Three studies (male-only cohort, n = 6–12) documented reversible post-race proteinuria, creatinuria and hematuria [25,32]. Elevation in hepatic enzymes AST and ALT were reported but remained below harmful thresholds and normalized within 48 h [30].

Four studies documented respiratory conditions in triathletes, with documented cases of asthma, airway hyperreactivity and swimming-induced pulmonary edema (SIPE) [37,41,42,51]. SIPE occurred in approximately 1.4% across different race distance (n = 749 male, 669 female) and was associated with hypertension, cold-water exposure, fish-oil intake and female sex. Symptoms included dyspnea, reduced oxygen saturation and auscultatory crackles [42]. A case study of a female athlete underlined the cold-water exposure [51] and use of 50 mg Sildenafil was in one cohort mentioned (sex not reported, n = 71) [41]. Airway hyperresponsiveness and asthma were more common in endurance triathletes (sex not reported, n = 30) [37].

Four studies responses cardiac responses and reported transient reductions in systolic performance following IRONMAN® 70.3 were demonstrated [43–45,52]. In one cohort (n = 9 male, 8 female), only male triathletes required higher dobutamine doses post-race to achieve an increase of 25 beats/min in heart rate or 10 mmHg/cm² in contractility, indicating reduced β-adrenergic responsiveness [52]. Three additional studies (male-only cohort, n = 9–14) reported no late potentials, and elevated troponin concentrations returned to baseline within 48 h [43–45].

Oxidative stress and inflammation responses increased after competition. Across ten studies (n = 6–26 male, 3 female and 13–57 sex not reported), oxidative stress markers (oxygen radicals, DNA breaks) and inflammatory cytokines (IL-6, IL-10, TNF-α) increased post-race and returned to baseline within 48 h [23–26,28–30,32,34,35]. Vitamin C and E supplementation showed no reduction in oxidative stress responses [24]. Experienced athletes demonstrated stronger oxidative and inflammatory responses compared to less trained individuals [26].

### Race related complications and medical strategies

Twenty studies reported race-related complications in IRONMAN® 70.3 (Tables 2 and 3). Overall injury incidence was approximately 10% [39,53], with most events occurring after 6–7 hours of racing [39,53]. The most common reported issues included hyperthermia and dehydration in one race [39], while another study identified gastrointestinal complaints (27.6%), musculoskeletal injury, and myalgia (25.4%) as the predominant issues [54].

**Table 3:** Medical support.

Study Participants	Title, Authors, Year	Result	Reference
n = 1 F	Swimming-induced pulmonary edema in a triathlete on active duty Haran et al. 2020	• The swimming-induced pulmonary edema was triggered by immersion in cold water with intensive physical exertion. • It is suspected that it is a disorder of the capillary-alveolar interface. • The therapy should consist of keeping the patient warm and dry, and also providing oxygen.	[51]
Overview	Medical considerations in triathlon competition Dallam et al. 2005	• The injury rate increases with increasing distance; In the half triathlon it reaches 10%. • Deaths occur due to bicycle accidents, accidental drowning, and cardiovascular disease. • Possible injuries include: hyperthermia or hypothermia, Dehydration fluid and electrolyte imbalance, respiratory diseases such as pneumothorax or bronchospasm, Anaphylaxis Arrhythmias drowning or near-drowning, injuries, musculoskeletal trauma, Stings and bites.	[39]
n = 575 N/A	A Temporal Model for Injuries in Non-Elite Triathlon Races Rimmer et Coniglione 2012	• In contrast to the Ironman, the prevalence of injuries in the Half Ironman shows 26.9% less, namely 10.8%. • 72.2% of injuries to amateur triathletes occurred in hours 6 and 7. • In contrast to the Ironman, the treatment time is shorter and the injuries are less serious.	[53]
n = 1,923 N/A	Patient presentations and medical logistics for triathlons over the full and half distance of Ironman Turris et al. 2017	• Of the 181 injured participants, 27.6% had gastrointestinal problems, 25.4% had musculoskeletal injuries, 20.4% had non-specific dizziness, 11.1% had fatigue, and 15.5% had other problems such as hyponatremia or bladder problems. • Most patients had already completed about 5.5 races. • Three patients were referred to hospital due to syncope, wound care and hyponatremia.	[54]
n = 187 N/A	Medical support during an Ironman 70.3 triathlon race. Yang et al. 2017	• There was one calf cramp during the swim, 19 injuries during the bike race, five of which required hospitalization, and 159 injuries during the run. • 127 of the injured showed myalgia	[55]
n = 750 N/A, HIR 17 N/A	Adherence to Follow-Up Recommendations by Triathlon Competitors Receiving Event Medical Care Joslin et al. 2017	• Of the triathletes who received medical care during the race, 2 in 17 (12%) sought follow-up care within a month of the race. • 4.6% of participants sought medical attention due to injuries.	[56]
n = 1 F	Deep venous thromboembolism in a triathlete Tao et Davenport 2010	• Due to bradycardia and the generally common musculoskeletal pain, deep vein thrombosis can be missed in healthy and young triathletes with no history. • The risk increases due to immobilization of driving to race, oral contraceptives in women, muscle hypertrophy and dehydration.	[57]
n = 289 M, 123 F, HIR 87 N/A	Hematocrits of triathletes: Is monitoring useful? O’Toole et al. 1999	• The mean hematocrits of 70 men are significantly higher and differ between different breeds. 17 women showed a lower hematocrit and no dependence on the race track. • The hematocrit was not age-dependent, men r = 0.20 and women r = 0.21. In women, there was a significant mean hemoconcentration, although there was generally high variability between participants. • There was no hematocrit HCT > 55% and the increase is explained by a training-induced higher increase in plasma volume, which correlates with V/O2max, than in red blood cell volume due to hemodilution.	[58]
n = 43 M, 5 F, HIR 28 M, 1 F	High-impact sport after hip resurfacing: The Ironman triathlon Girard et al. 2017	• After 4.7 years, there were no cases of dislocations or implant revisions. • Of the 29 IRONMAN 70.3^®^ patients, 21 again ran a half triathlon with an unchanged mean power P = 0.4. The surgical technique consisted of a posterolateral approach, to the extent of a capsulotomy	[59]
n = 1 M	Clavicle Fracture and Triathlon Performance: A Case Report Gerdesmeyer et al. 2024	• An triathlete was able to run a half triathlon 3 weeks after a broken collarbone that had to be operated on. • He received therapy with extracorporeal magneto-transduction plus physiotherapy for 30 minutes at 8 Hz and a magnetic field strength of about 80 mT every other day for 2 weeks.	[60]
n = 1,285 N/A	Risk of Exercise Addiction: A Comparison of Triathletes Training for Sprint, Olympic, Half Ironman and Ironman Distance Triathlons Youngman 2007	• 20% of triathletes show a tendency towards training addiction, which increases with longer distance races, although in the half triathlon there was an outlier upwards at 4 cells. • Training 10 hours a week or more shows a higher risk of exercise addiction. • There is no significant association between sex and sports addiction risk, with a moderate level of addiction in men being slightly increased at 81.2% to 77.6% and a high addiction potential in women at 17.9% to 21.7% across all disciplines.	[61]
n = 1 M	Physiological Changes Across a Sport Season in a Nine-Time World-Champion Triathlete: A Case Report Gonzalez-Custodio 2025	• Measurements were taken after a 3 month break due to injury, during training and at peak times. • Body fat percentage fell by 3.1% and skinfold thickness decreased by about half. • Ventilatory efficiency and altered body mass are considered the main mechanisms for recovery with a win at the Ironman 70.3 World Championship	[62]

Complications varied by race segment. Injuries occurred most frequently during the running segment, followed by cycling, and least often during swimming [55]. Swimming-related risks included drowning and swimming-induced pulmonary edema [39,51], while cycling incidents often involved trauma requiring medical intervention [54,55]. Severe outcomes were rare, with a fatality rate <0.01%, primarily due to cycling accidents, drowning, or cardiovascular events [39], with one report estimating a cardiovascular death rate of approximately 1 per 50,000 athletes [39].

Medical support and clinical outcomes were investigated in several studies. Hospitalization was required in 7.5–12% of injuries, most commonly for syncope, wound care, or hyponatremia [54,56], but only 2 of 17 athletes sought follow-up care [56]. Deep vein thrombosis was documented in a case report, potentially linked to bradycardia or contraceptive use [57]. Monitoring of hematocrit (n = 70 male, 17 female) proved unreliable, as values fluctuated without exceeding the clinical threshold of >55% [58]. 21 of 29 (sex not reported) triathletes demonstrated successful completion of IRONMAN® 70.3 with prosthetic joints [63], and rehabilitation after a fracture appeared to benefit from extracorporeal shock-wave therapy in a case report [60].

Preventive and organizational factors were also reported. Sun exposure reached 1.2 SED/h during cycling and 3.0 SED/h during running, exceeding the minimum erythema dose of approximately 1.0 SED for fair-skinned individuals up to 5.0 SED for dark-skinned individuals [64]. Compression stockings did not reduce medical complications (male-only cohort, n = 36) [46], while ulnar neuropathy appeared frequently but without long-term sequelae (sex not reported, n = 777) [47]. Structured on-course medical systems reduced treatment times and improved outcomes in IRONMAN® 70.3 [39,53]. Most studies focused on physiological aspects, however one psychological study reported increased maladaptive or addictive training behavior in individuals training more than 10 h·week ⁻ ¹ and in triathletes competing in longer events [61].

### Performance determinants and race characteristics

Twenty-five studies investigated predictors of IRONMAN® 70.3 performance, including environmental and race-related factors (Tables 4 and 5) [8–10,13,14,66–74,81]. In large overview studies (both sex, n = 8,340–16,611), the primary determinants included driving dynamics, environmental conditions, competitor density, course topography, age and sex [10,68,69,73]. Across IRONMAN® 70.3, cycling consistently emerged as the strongest predictor of overall performance [10,13,14,68,69,73], accounting for 50–60% of race duration [1,82], correlations with finishing time reached 0.85. Running showed correlations of 0.75 in women and 0.82 in men, while swimming correlations were 0.46 and 0.63, respectively [73]. V̇O₂max explained 67% of the variance in overall race time [74].

**Table 4:** Performance analysis.

Study Participants	Title, Authors, Year	Result	Reference
n = 10,176 M, 6,491 F, HIR 8,340 M and F	What is the best discipline to predict the overall performance of a triathlon? An analysis of Sprint, Olympic, Ironman^®^ 70.3 and Ironman® 140.6 Sousa et al. 2021	• Cycling is the best predictor of total race time in the 70.3 for professional triathletes because it represents the larger portion of the race distance.	[10]
n = 16 M	Association between body composition and explosive strength in middle distance amateur triathletes Garcìa-Chavez et al. 2023	• Recreational triathletes can benefit from strength training. • A significant correlation was found between body mass index and countermovement performance. • A significant correlation was found between muscle mass and bone mass to squat jump performance.	[65]
n = 858 M, 380 F	Pacing in ultra-endurance sports: Investigating the pacing differences between gender, age and target performance during the Muskoka Ironman 70.3 triathlon E. Watters 2019	• In cycling, the speed from the first to the second half increased by 1.14 km/h (p = 0.002) for men and 1.19 km/h (p = 0.009) for middle-aged triathletes (35–45) by 1.19 km/h (p = 0.009). • All groups had a slower second half of the run, with the top performers (25%) getting less slower than the taillights (75%) (0.37 km/h vs. 0.60 km/h, p = 0.003). Men, lower-performing and middle-aged triathletes slowed down their performance more in the second half.	[66]
n = 2 F, 3 M	Differences in the bioelectrical activity of the major muscle groups of the lower limb using road or triathlon position Drozdek et al. 2014	• During the pressure phase, the working muscles in the TRI position showed increased activity. • In muscle activity there were no significant differences when pedaling between the ROAD and TRI positions.	[67]
Overview	Factors that affect the pace in triathlon Wu et al. 2014	• The biggest factors influencing the total duration are driving dynamics such as environmental factors, other competitors and topography, as well as age and sex. • For overall speed, a conservative pace strategy that allows glycogenic reserves to be preserved may be better	[68]
n = 16,611 N/A	A machine learning approach to find the fastest race course for professional athletes competing in Ironman® 70.3 between 2004 and 2020 Thuany et al. 2023	• The fastest results were observed in the races with the most participants. • The race of the Ironman® 70.3 World Championship brought the fastest results.	[69]
n = 8 M	Pacing strategies during the swimming, cycling and running disciplines of sprint, Olympic and half Ironman triathlons Wu et al. 2015	• The triathletes in a sprint, Olympic and half triathlon used a consistent swim pace strategy, while the cycling and running disciplines showed lower power output and higher heart rates in the half triathlon.	[70]
n = 150 M / 150 F	Changes to the transition periods at the ‘Ironman Hawaii’ between 1998 and 2013 Rüst et al. 2014	• The 10 fastest men and women in the half triathlon were able to reduce the transition times, and the sex difference remained unchanged. • The transition times in the half triathlon were faster than in the triathlon.	[71]
n = 625,398 M, 198,066 F	Modeling Performance in IRONMAN^®^ 70.3 Age Group Triathletes Thuany et al. 2025	• The sex difference was between 12–18% among amateur triathletes. • The best performances were shown by Danish and Swiss amateur men. • The best performance among amateur triathletes could be observed in St. Pötel Austria.	[72]
n = 10,282 M, 6,329 F	Cycling is the main predictive split discipline among professional Ironman® 70.3 triathletes Weiss et al. 2024	• The strongest correlation to the final time was shown by cycling (0.85), followed by running with 0.75 for women and 0.82 for men. Swimming shows a moderate correlation of 0.46 for women and 0.63 for men.	[73]
n = 6 M, 6 F	Key factors influencing cycling performance and overall race time in the Ironman 70.3 for amateur athletes Vivian et al. 2024	• The FTP / Functional Threshold Power can predict 63.8% of the cycling time, as the most important predictor of overall performance; it is calculated from the fat and muscle percentage. • Muscle mass predicts 77.7% of FTP. • This study also showed that VO2 max is the best predictor for all three disciplines and is calculated via fat mass.	[74]
n = 16 M	Relationship between body composition and physical performance in amateur middle-distance triathletes Manrique Lenis et al. 2025	• Muscle strength correlates significantly with swimming and running performance. • The wheel performance correlates with the performance in the jump tests, as well as with less fat mass.	[75]
n = 60 N/A	Modeling preference time in middle distance triathlons Fister et al. 2017	• An AI-powered tool that uses the Particle Swarm Optimization (PSO) algorithm to calculate ideal times for swimming, cycling, running, and transitions. • It works with Python, using triathletes’ best times. • The higher the correlation in the historical data, the more reliable the prediction.	[76]

**Table 5:** Environmental conditions and performanceperformance.

Study Participants	Title, Authors, Year	Result	Reference
n = 8 N/A	Measured UV exposures of Ironman, sprint and triathlon participants over the Olympic distance Downs et al. 2020	• The swimming could not be measured due to a defective device. Cycling showed a 3.1 standard erythema dose/SED (2 h 38 min) or 1.2 SED/h running a 4.8 SED (1 hour 36 minutes) 3.0 SED/h • The data was measured outside the summer in Australia.	[64]
n = 852,721 N/A	A descriptive analysis of the fastest race courses for triathletes. Thuany et al. 2024	• The Ironman® 70.3 Zell am See (Austria) showed the fastest overall time. • The Ironman® 70.3 European Championship Elsinore and the Ironman® 70.3 World Championship are the fastest courses for the top 100 triathletes.	[77]
n = 146,924 N/A	Performance comparison of long-distance running competitions under different meteorological and environmental conditions. D. Berke 2019	• In Hungary, the pace decreased as the temperature (>25°C) and humidity (>70%) as well as the absolute altitude were higher. • The optimal temperature for triathlon runners is around 5–7°C.	[78]
n = 34 N/A	Relationship between physiological parameters and performance in a half-Ironman triathlon in the heat Del Coso et al. 2014	• Faster triathletes experienced a greater reduction in body mass of −4.1% and higher osmolality, coupled with a higher core temperature of +1.3 degrees Celsius after the race. This suggests that exercise intensity was the main reason for the increase in core temperature. • Slower triathletes with a deeper dehydration of −2.8% body mass showed a higher degree of muscle damage and decreased muscle performance.	[48]
n = 19 M	Hydration and thermoregulation during a half-Ironman in a tropical climate Baillot et Hue 2015	• Performance is not related to the degree of dehydration in acclimatized triathletes. • The ad libitum fluid intake is considered functional for the acclimatized sports group.	[79]
n = 12 N/A	Long-distance travel to the northeast disrupts sleep and leads to perceived fatigue in endurance athletes Stevens et al. 2018	• Long-distance travel to a race in a hot environment after a cool winter showed reversible negative effects on sleep and fatigue in the first 48 hours. • The immunity of the mucous membranes and physiological stress, as measured by sIgA and cortisol, were not negatively affected. Although three triathletes reported symptoms of illness 3–5 days after their arrival.	[49]
Overview	Factors Influencing Amateur Triathlon Participation in South Korea: A Comparative Study Based on Experience Levels C. G. Lee 2025	• Depending on your experience level, their motivations for half triathlon vary. • For beginners it is a challenge and experienced triathletes focus on health and fitness. • Time management and physical demands are the biggest hurdles for participants. • Ideas for lowering the barriers include more social support and media exposure for beginners, as well as more events in major cities in South Korea.	[50]
Overview	Images of the destination projected in the sport event website: A case of Ironman 70.3 Qujing L. Yan 2019	• The study shows that the projected finish images from Ironman 70.3 Qujing and other sporting events differ from the actual finish images and that sports organizers should change this in order to promote the destination more effectively.	[80]

sIgA means saliva immunoglobulin A.

Performance strategies were examined in relation to race execution, pacing, and biomechanics. Analyses (n = 858 male, 380 female) revealed speed declines of 1.14 km/h in the second half of cycling and 0.37 km/h (professionals) to 0.6 km/h (amateurs) in running, with larger decrements observed in men, middle-aged, and lower-performing triathletes [66]. A conservative pacing strategy with a glycogen preservation were detected as particularly important for maintaining running performance in the final race segment [68].

Biomechanical and technical factors further modulate performance outcomes. In a small cohort (n = 3 male, 2 female), the TRI position was associated with optimized force application during cycling [67]. Strength training has been shown to produce better results (male-only cohort, n = 16) [65,75]. Transition efficiency, which improved among top-10 finishers over time, although sex differences in transition times persist across age groups [71,63]. Swim pacing remained relatively stable across race distances and was associated with overall performance in amateur but not professional triathletes [81,70].

The race venue and environmental conditions were associated with performance results. The fastest top-100 IRONMAN® 70.3 performances were observed at the World Championship and the European Championship in Elsinore [69,77], while the fastest individual split occurred at Zell am See [77]. Among male amateurs, peak results were recorded in St. Pölten [72], and triathletes from Belgium, Denmark and Switzerland achieved the best outcomes across events [7]. Environmental load exerted a measurable influence on performance in IRONMAN® 70.3, with speed declined at temperatures exceeding 25 °C, humidity above 70%, and at altitude [78,48]. Optimal running temperatures were estimated at 5–7 °C (both sex, n = 146,924) [78], consistent with fast performances reported in Zell am See (both sex, n = 852,721), where race-day temperatures ranged from 11–20 °C [77]. Heat-acclimatized triathletes (male-only cohort, n = 19) showed attenuated performance impairments [79], while faster triathletes (sex not reported, n = 34) demonstrated greater reductions in plasma osmolality under heat stress [48]. Travel from cold to warm climates impaired sleep but normalized within 48 hours without inducing immune suppression (sex not reported, n = 12) [49].

Environmental and organizational factors contributed to the race outcomes. Social-environmental factors, including media exposure, urban race settings and structured event organization, were associated with increased novice participation in South Korea (sex not reported, n = 200) [83], furthermore, it was shown that promotional race material often differed from actual environmental conditions [80]. Course topography contributed to split-time variance in cycling and running [67,68,84], and higher competitive density corresponded with faster aggregated field results in professional triathletes [69,72].

### Nutrition strategies

Ten studies examined race-day and preparatory nutrition for IRONMAN® 70.3 triathletes (Table 6). Higher carbohydrate intake was associated with improved performance across studies [85–89], and IRONMAN® 70.3 triathletes consumed more carbohydrates and water than full-distance IRONMAN® triathletes without reporting increased gastrointestinal symptoms (both sex, n = 221) [89]. Sodium supplementation was beneficial for triathletes with high sweat loss [22,90]. During an IRONMAN® 70.3 race, essential amino acids decreased by 27.1%, branched-chain amino acids (valine, leucine, isoleucine) by 32.8%, and non-essential amino acids by −24.4%, with no correlation to fatigue [23]. Dietary interventions were limited. However, a case report describing a 32-week low-carbohydrate, high-fat diet led to the worst recorded performance including depression and irritability [91].

**Table 6:** Nutritional strategies.

Study Participants	Title, Authors, Year	Result	Reference
n = 15 N/A, HIR 10 N/A	FEEDING, HYDRATION AND SUPPLEMENTATION STRATEGIES, IN THE TRAINING AND COMPETITION TIME OF TRIATHLETES AT THE IRONMAN 140.6 AND 70.3 BRAZIL. Datsch Benneman et al. 2018	• Deficiencies in vitamin D, potassium, calcium 60% (n = 6), zinc 40% (n = 4), magnesium 40% (n = 4) and vitamin A 30% (n = 3) were found. • Branched-chain amino acids and carbohydrates were the most commonly used ergogenic nutrients.	[85]
n = 1 M	Muscle Glycogenolysis and Resynthesis in Response to a Half-Ironman Triathlon: A Case Study Gillum et al 2008	• Muscle glycogenolysis is crucial for finishing the race and preventing muscle damage. Therefore, exogenous carbohydrate intake is important during the race.	[86]
n = 74 M	Multiple-Transportable Carbohydrate Effect on Long-Distance Triathlon Performance Rowlands and Houltham 2017	• Multi-transportable 2:1 maltodextrin/glucose-fructose carbohydrates show a slight performance advantage of 0.5% over easily transportable carbohydrates.	[87]
n = 84 M, 32 F, HIR 65 M and F	Many non-elite multisport endurance athletes don’t follow sports nutrition recommendations for carbohydrates Masson et Lamarche 2016	• Of the recreational triathletes, 86% consumed at least 1.2 g of protein per kg and 45% reported consuming at least 1.6 g of protein per kg. 27% consumed at least 6 g/kg of carbohydrates. • However, at the World Cup, 70% consumed at least 1.6 g/kg of protein and 40% consumed at least 6 g/kg. • No difference between the sex could be detected.	[88]
n = 221 N/A	Food intake and gastrointestinal problems in endurance competitions Pfeiffer et al. 2012	• Compared to a full triathlon, participants in the 70.3 consumed 65 ± 25 g/h of carbohydrates, which is + 30 g/h above the triathlon. • Fluid intake was also increased to 794 ± 339 ml/h, compared to 354 ± 187 ml/h for the entire triathlon. • 14% had gastrointestinal problems that did not correlate with carbohydrate intake. • The study works with an unknown cohort size for the half triathlon and the individual diet is very variable.	[89]
n = 26 N/A	Effects of Oral Salt Supplementation on Physical Performance During a Half Ironman: A Randomized Controlled Trial Del Coso et al. 2016	• The use of salt shows a positive effect on overall performance 333 vs. 307 min., rehydration and reduction of muscle fatigue. • 113 mmol Na+ and 112 mmol Cl− were given in the salt group. Exact information about the amount could not be obtained from the study.	[90]
n = 1 M	Case Study: Long-Term Low-Carb, High-Fat Diet Impairs Performance and Subjective Well-Being in a World-Class Long-Distance Vegetarian Long-Distance Triathlete I Mujika 2018	• The long-term (32 weeks) low-carb, high-fat (LCHF) diet led to the lowest rankings in the triathletes in half Ironman and Ironman races. • During the LCHF diet, the triathlete reported depression, irritability and low mood. The diet also didn’t solve the gastrointestinal problems he had at Ironman races.	[91]
n = 20 F	A Preliminary Study on Nutrients Associated with the Risk of Relative Energy Deprivation in Sport (RED-S) in High-Performance Amateur Triathletes: Results of a Nutritional Assessment Langa et al. 2025	• High-performance triathletes from different breeds were examined • Fewer menstrual cramps, gastrointestinal discomfort and injuries were found in diets with more animal protein and a higher proportion of saturated fat to unsaturated fatty acids.	[92]
n = 11 M	Creatine supplementation reduces plasma levels of pro-inflammatory cytokines and PGE2 after a half-Ironman competition Bassit et al. 2007	• Experienced triathletes took 10 g of creatine twice a day for 5 days and compared it to a control group. • At 24 hours after the race, the creatine group showed an 85.5% or 48-hour reduction in PGE2 by 91%, TNF-alpha was 42% and 64% lower, and INF-alpha was 50.5% and 80.1% lower, respectively, compared to the control group. • Interleukin 6 showed no differences.	[93]
n = 2,576 M, 411 F	Refined analysis of a doping cross-sectional survey among recreational triathletes: support for the gateway hypothesis of dietary supplements. Heller et al. 2020	• The probability of using doping substances within one year is significantly higher among amateur triathletes who consume NS (20.6%) than among non-doping triathletes (11.4%). • Concepts for doping prevention should therefore start with the intake of dietary supplements.	[94]

PGE2 stands for prostaglandin E2, TNF-alpha stands for tumor necrosis factor-alpha and INF-alpha stands for interferon-alpha.

Among twenty elite premenopausal female triathletes, higher intake of animal and saturated fats was associated with a lower incidence of relative energy deficiency in sport (RED-S) [92]. One study (male-only cohort, n = 11) found reduced post-race immunosuppression after creatine supplementation for 20 g/day for 5 days [93]. Finally, supplement use was associated with a risk of inadvertent doping [94].

### Peak performance and participation trends

Ten studies investigated age-related performance patterns in IRONMAN® 70.3 across large datasets ranging from approximately 5.000 to over 800.000 male and female triathletes (Table 7). Peak overall performance occurred in men aged 18–39 years and women aged 25–39 years [3–5,75,95]. Stones and Hartin reported peak performance at 34 years in men and 35 years in women [3,4,75], while amateur triathletes performed best between 25–29 years [72]. Longitudinal data indicated a U-shaped performance curve in men but not in women, with the largest discrepancy between 18–24 and 45–54 years [3,5,75]. Performance decline began earlier in women (from 30–34 years) than in men (from 35–39 years) for both amateur and professional triathletes [3–5].

**Table 7:** Age-related difference in performance.

Study Participants	Title, Authors, Year	Result	Reference
n = 3929 M, 1620 F	Aging and Half-Ironman Performance M.J. Stones et A Hartin 2017	• A cross-sectional decline was observed in the age group of 35–39-year-olds of professional triathletes. • Among 35–39-year-olds, running times increased. Swimming times increased among 50–55-year-olds. The travel times remained the same.	[3]
n = 206,524 F, 633,576 M	The age-related drop in performance at the Ironman 70.3 Jäckel et al. 2020	• There is a general decline in performance among amateur triathletes between the ages of 30 and 34 for women and 35–39 years for men. • There were hardly any declines in cycling. • In running, there was a decline from the age of 26 for men and 28 for women. • Swimming was the strongest predictor of a drop in performance in women aged 35–49 and men aged 40–54.	[4]
n = 1115 F, 5188 M	Age and gender differences in half Ironman triathlon performances – the Ironman 70.3 Switzerland from 2007 to 2010 Knechtle et al. 2012	• The best results were achieved by women between the ages of 25 and 39, and men between the ages of 18 and 39. • The smallest age-related decline was observed in cycling	[5]
n = 146,924 N/A	Performance comparison of long-distance running competitions under different meteorological and environmental conditions. D. Berke 2019	• Medium runners (5:00–6:59 min/km) increase continuously. • The 30–49-year-old triathletes have gained significant weight between 1997 and 2017 and improved their performance. • The number of participants +50 slowly increases.	[78]
n = 14,592 M and F	The best triathletes are older for longer race distances – a comparison between triathlon over the Olympic, half-Ironman and Ironman distances Knechtle et al. 2014	• A reduction in maximum oxygen consumption (VO_2_max) and lactate threshold appear to be the main causes of age-related decline in performance. • Muscle atrophy from the age of 50 can be compensated for by hypertrophy. • The decline in performance begins at the age of 40 when swimming and cycling, and at the age of 45 when running. • The Olympics have the youngest participants, followed by the triathlon over the half Ironman distance and then the Ironman distance	[95]
n = 625,393 M, 198,066 F	Comparing the Performance Difference Between Men and Women in the Older Age Groups at the IRONMAN® 70.3: An Internet-Based Cross-Sectional Study of More Than 800,000 Race Records Knechtle et al. 2023	• The age group of 35–39-year-olds was most strongly represented. • The number of participants fell most sharply in the age group of 50–69-year-olds. • The number of participants in the 70–74 and 75 + age groups is higher in this study.	[96]
n = 198,066 F, 625,393 M	Prediction of the overall performance of the Ironman 70.3 age group triathletes through split disciplines Nikolaidis et al. 2023	• In the linear regression model for completion time, the coefficients of cycling time 0.759 and transit times 0.805 are much larger than the swimming time coefficient 0.394. The difference in swimming performance between women and men is small and reduces for women aged 45 and over and for men aged 35 and over. • The difference in performance between men and women decreases from the age of 50.	[97]

Discipline-specific analyses showed peak performance at different ages: in swimming (28 years women, 26 years men), cycling (35 years women, 34 years men) and running (31 years women, 32 years men) [3,4]. The age-related performance decline also differed by discipline, with swimming predicted performance reduction between 35–49 years in women and 40–54 years in men [4]. Running showed the steepest age-related drop, while cycling exhibited the smallest decline [4,5].

Eight studies analyzed sex differences in IRONMAN® 70.3 (Table 8). In the eight large overviews, women were underrepresented, reflecting lower female participation rates in IRONMAN 70.3 ® events. However, the top-performing studies showed a 1:1 ratio [63]. Women performed lower across all age groups, with narrowing sex gaps at older ages [3–5,72,95–97]. Nikolaidis et al. reported that the performance gap decreased from age 50 onward [97], while the largest sex difference among top 10 triathletes occurred in the 60–64 age group [63]. Swimming showed the smallest sex difference at ~2:13 minutes in the 18–24 age group, although a top-10 cohort showed a 37.5% difference in swimming [63,96]. In cycling, the smallest sex gap occurred between 70–74 years (~17:05 minutes) [96], and 11.2% in top triathletes [5,63].

**Table 8:** Sex-specific differences.

Study Participants	Title, Authors, Year	Result	Reference
n = 3929 M, 1620 F	Aging and Half-Ironman Performance M.J. Stones et A Hartin 2017	• Women performed best in the 35–39 age group, while men performed best in the professional triathlete age group between 18 and 39 years old. • The discrepancy is greater between 18–24 and 45–49 years.	[3]
n = 206,524 F, 633,576 M	The age-related drop in performance at the Ironman 70.3 Jäckel et al. 2020	• After a 35-year plateau, women show a greater decline in performance than men. • In 22 years, women have reduced the difference in performance compared to men from 15% to 11%. The strongest predictor of this is running.	[4]
n = 1115 F, 5188 M	Age and gender differences in half Ironman triathlon performances – the Ironman 70.3 Switzerland from 2007 to 2010 Knechtle et al. 2012	• When it comes to performance in cycling, the difference between the sex is small. • Significant sex differences in performance were observed between the ages of 18 and 24 and between the ages of 45 and 54.	[5]
n = overview	Women in the triathlon—the differences between female and male triathletes: a narrative review Loosli et al. 2025	• A steady increase in participation of male and female could be observed. • The sex difference was lowest in the split discipline of swimming, with cycling contributing the highest correlation to overall performance. • The peak performance age for women is 31.6 ± 3.4 years	[6]
n = 9 M, 8 F	Sex differences in left ventricular function and β-receptor responsiveness after prolonged strenuous exercise Scott et al. 2006	• After a half triathlon, men showed an increased amount of dobutamine to increase heart rate by 25 beats/min or increase contractility by 10 mmHg/cm^2. Chronotropic and inotropic sensitivity decreased in women. • The decrease in fractional area, diastolic filling, contractility and wall tension is higher in men than in women.	[52]
n = 90 M, 90 F, HIR 60 M and F	Age and sex differences pertaining to modes of locomotion in triathlon Stevenson et al. 2013	• The data of the top ten male and female amateur triathletes between 2009 and 2011 in the age groups between 18 and 64 years were examined. • The time differences were largest among 60–64-year-olds at 21.7%, and also the largest among swimmers at 37.5%. The smallest difference was observed in cycling compared to the short disciplines with 11.2%. The transition period was longer for women than for men.	[63]
n = 14,592 M and F	The best triathletes are older for longer race distances – a comparison between triathlon over the Olympic, half-Ironman and Ironman distances Knechtle et al. 2014	• Women seem to do best at age 32 and men at age 28.	[95]
n = 625,393 M, 198,066 F	Comparing the Performance Difference Between Men and Women in the Older Age Groups at the IRONMAN® 70.3: An Internet-Based Cross-Sectional Study of More Than 800,000 Race Records Knechtle et al. 2023	• The average difference between men and women was 9:47 minutes for running, 2:13 minutes for swimming and 17:05 minutes for cycling. • The sex-specific differences were smallest for running in the age group of 30–34 years old, for swimming in the age group of 18–24 years and for cycling in the age group of 70–74 years old. • The differences in performance between men and women decreased with age. Running after the age of 70, swimming after the age of 60 and cycling declined continuously.	[96]

Participation trends were reported in large datasets (both sex, n = 146,924–840,100). From 1997–2017, participation increased in both sexes, particularly among triathletes aged 30–49 years, with fastest improvements observed in runners over 40 years and more triathletes with paces of 5:00–6:59 min/km [78]. Participation also increased in triathletes >50 years, though more slowly [78]. However, male triathletes were more likely to complete the race than women [96]. Between 2004–2020, the 35–39 age group was the most represented, with 186 triathletes aged ≥70 years included [96].

### Performance predictors and training characteristics

Five studies examined training strategies and characteristics for IRONMAN® 70.3 (Table 9). Recreational triathletes complete 20–24 weeks of preparation with 6.5–11.5 h·wk ⁻ ¹ training [84,98]. Over this period, V̇O₂max increased by 2.7 ml/kg/min and maximal power by 14.6 W in triathletes aged 39 ± 9.9 years (n = 19 male, 13 female) [98], with greater improvements in triathletes with higher fat mass and more endomorphic somatotype (male-only cohort, n = 14 male) [84]. Cycling training showed the strongest association with overall performance and explained >50% of variance [84,98,99], whereas swimming contributed the smallest training portion and showed no transfer effects to cycling or running [84,98]. Zone-2 training improved running performance (male-only cohort, n = 18), whereas medium-intensity intervals did not affect total race time [81]. Standardized training load metrics (e.g., TSS, power-based data) were largely absent, limiting comparability between studies [81,84].

**Table 9:** Training program.

Study Participants	Title, Authors, Year	Result	Reference
n = 18 M	Polarized and pyramidal training intensity distribution: Relationship with a triathlon competition over the half-Ironman distance Selles-Perez et al. 2019	• Training with the higher percentage of 18.8% to 4.3% in the Zone 2 ventilation threshold showed better running speed. • Swimming performance correlates most strongly with total time. • For the total time, experience, total training volume and body composition are more important than training in the different ventilation thresholds	[81]
n = 14 M	Changes in triathletes’ performance and body composition during a given training period for a half Ironman race Sellés-Pérez et al. 2019	• Amateur triathletes benefited the most from 13 weeks of training, with higher percentage fat mass and endomorphine levels and lower ectomorphism. • VO_2_max levels increased more when cycling than when running. More intense cycling training improved mileage and overall performance due to reduced fatigue.	[84]
n = 19 M, 13 F	Preparation for an Half-Ironman^tm^ Triathlon amongst Amateur Athletes: Finishing Rate and Physiological Adaptation Lalonde et al. 2020	• A 24-week training plan for amateur triathletes seems ideal. 31 of 32 participants crossed the finish line. • The training lasted between 6.5 and 11.5 hours per week • Swimming was the least exercised because it had little impact on the final result or there was a lack of opportunities • The maximum oxygen consumption increased by 2.7 ml/kg/min, the maximum power by 14.6 W and the absolute oxygen consumption and the power of the ventilation thresholds also increased by 0.3 l / 9.9 W.	[98]
n = 155 M, 54 F	Predictive Variables for Performance in the Half Ironman Triathlon Gilinsky et al. 2014	• Anthropometric features such as a lower body fat percentage, fewer rest days and more races showed better results. • Psychological aspects such as lower race time goals and a greater will to reach the finish time showed better overall performance • The strongest predictors, which accounted for 58% of the variance, were the best time of the Half Ironman race, the age, goal setting, and success time of the finish.	[99]
n = 200 M, 168 F	The influence of triathletes’ serious leisure traits on sport constraints, involvement, and participation Ma et al. 2022	• Recreational triathletes between the ages of 22 and 71 and a similar sex distribution were examined. • Triathletes with serious recreational characteristics such as endurance, effort invested, benefits, ethos, and identity show a positive correlation with daily training and distances traveled.	[100]

In addition to training characteristics, several predictors were associated with training outcomes and race performance. Anthropometric factors, especially lower body fat percentage in men, training experience, total body volume, and balanced rest days predicted faster race times (n = 155 male, 54 female) [99]. Personal goals, previous race times, and goal commitment were additional predictors [99]. Endurance-related traits such as ethos and identity improved adherence to training (n = 200 male, 168 female) [100].

### Synthesis of results

**Table:** 

Summary points
1. Growing participation with increasing representation of women and older triathletes.
2. Peak performance typically in men aged ~18–39 and women aged ~25–39.
3. Cycling as the strongest predictor of total race time across large cohorts.
4. Effective preparation spanning 20–24 weeks with ~6.5–11.5 h·wk ⁻ ¹ and race-day strategies emphasizing carbohydrate availability and individualized sodium and water replacement.
5. Race-related physiological responses marked by transient immunosuppression and body-mass changes.
6. Performance decrements under heat, humidity, and altitude.
7. Medical encounters clustering in the final race segment.

## Discussion

To date, no review has synthesized evidence specific to IRONMAN® 70.3. This work consolidates data broken down by physiological adaptations, competition-related complications and medical strategies, performance determinants and competition characteristics, nutritional strategies, age and gender in peak performance, as well as training characteristics and performance predictors.

Comparative analyses with other race formats, such as the full-distance IRONMAN® and the Olympic-distance triathlon, were used to identify IRONMAN® 70.3-specific characteristics and generalizable findings. However, comparability across studies was limited by substantial data heterogeneity, including non-standardized performance metrics, variable environmental conditions, and inconsistent reporting practices. Outcome definitions and measurement protocols differed considerably, particularly regarding the timing of physiological assessments and the distinction between in-race and post-race data.

Many studies relied on small sample sizes, predominantly consisting of young to middle-aged male triathletes, which restricts generalizability to female triathletes, older adults, and recreational participants. This male bias is further reinforced by participation patterns and the limited availability of female-specific cohorts. Additionally, studies often failed to stratify results by competitive level.

Methodological limitations also include the frequent reliance on cross-sectional designs and the scarcity of controlled interventional studies, both of which constrain causal inference. Environmental factors such as temperature, humidity, altitude, and course topography were inconsistently reported, further limiting contextual interpretation. In some cases, identical participant cohorts were reused across multiple publications, introducing a risk of unit-of-analysis bias. Furthermore, medical outcomes were often based on self-report, increasing susceptibility to recall bias.

To strengthen future evidence, research should prioritize:

- longitudinal monitoring of training load and recovery biomarkers in both sexes
- controlled intervention studies on nutritional strategies and heat acclimation
- increased inclusion of female triathletes and older adults
- standardized in-race physiological monitoring using wearable technologies

These approaches would enhance mechanistic understanding and enable more precise, sex- and age-specific recommendations for triathletes and practitioners.

### Physiological responses

This summary shows that the IRONMAN® 70.3 induces marked but short-lived alterations in hydration status, electrolyte concentrations, oxidative and inflammatory activity, muscle-damage biomarkers and renal and hepatic indices [20–35]. Despite the magnitude of these disturbances, most parameters normalize within hours until 48 hours and no values exceeding the pathological threshold [25,27,30,34,35], underscoring robust homeostatic recovery mechanisms in trained triathletes. This rapid resolution mirrors patterns observed in other endurance modalities but appears less pronounced than in full-distance IRONMAN® events, consistent with a lower cumulative load and a shorter duration of exertion [101,102].

A reduction in fat mass combined with a significant increase in circulating free fatty acids suggests a shift toward a lipid-dominated metabolism, which is characteristic of prolonged moderate-intensity exercise [33]. In contrast to full-distance IRONMAN®, with a reported skeletal-muscle loss [33], muscle mass is preserved during the IRONMAN®70.3 [33,103], likely due to the shorter Olympic-distance events [103,104].

Preservation of muscle tissue aligns with sustained reliance on lipid oxidation at moderate exercise intensities and with the shorter total race duration, which reduces cumulative catabolic strain [103].

Elevated intracellular and total body water before race have been associated with superior performance [31,105], although interpretation should consider the susceptibility of bioimpedance techniques to acute fluid shifts [48]. Age-related differences in anthropometry, ranging from ectomorphic profiles in younger triathletes to increased endomorphy in triathletes above 35 years, reflect long-term morphological adaptations [106]. Amateurs consistently display higher fat mass and lower body water percentage, than professionals [5,6,48]. This patterns are consistent with training induced plasma volume expansion and metabolic adaptations [5,6,48].

Electrolyte changes were modest and remained within physiological rages. The decline in sodium without clinical hyponatremia support individualized sodium and fluid strategies during exercise [22,107]. Evidence for supplementation other electrolytes remains inconclusive, due to variability of sweat rate, environmental conditions and personal intake [20–22,34,108].

A sharp increase in creatine kinase, myoglobin, and lactate dehydrogenase following competition indicates muscle stress caused by physical exertion; however, these levels return to normal within 48 hours, suggesting a reversible process [25,27,34,35,109]. Biomechanically, temporary disturbances in Ca² ⁺ homeostasis, the formation of reactive oxygen species (ROS), and increased free fatty acids contribute to membrane instability without causing structural damage [101,102]. The lower magnitude compared to endurance competitions supports a classification as moderate systemic stress within the context of adaptability [33]. An increase in biomarkers of muscle damage is therefore best interpreted as temporary permeability of the sarcolemma membrane rather than structural myofibrillar damage [25,27,34,35].

Temporary proteinuria, creatinuria, and hematuria caused by physical exertion reflect glomerular permeability and renal hemodynamics [25,32,110,111]. Elevated AST and ALT levels, which normalize within 48 hours, are more indicative of functional enzyme release from muscle and liver than of hepatocellular damage [30,112,113]. These patterns are consistent with those seen in other endurance sports and suggest transient organ stress with rapid recovery [112].

The increase in markers of oxidative stress and cytokines (IL-6, IL-10, TNF-α) reflects the formation of reactive oxygen species (ROS) and immune activation during prolonged exercise [23–26,28–30,32,34,35]. These reactions are not exclusively harmful but serve as signals for repair mechanisms [26,29,30,32,34,35]. Stronger reactions in experienced athletes likely reflect a higher absolute training load, not maladaptation [26]. Vitamin C and E supplementation showed no reduction in these markers [24]. This is consistent with the finding that exogenous antioxidants do not attenuate the oxidative signaling triggered by physical activity [24].

The higher prevalence of airway hyperresponsiveness, asthma, and SIPE suggests a susceptibility of the airways in endurance triathletes [37,41,42,51,114–118]. Respiratory conditions, including asthma and airway hyperreactivity, remain relevant due to exposure to environmental irritants, airway drying and high ventilatory flow [114–116]. SIPE is associated with various pathophysiological factors, such as cold-water exposure, hypertension, and female gender. This underscores the involvement of hemodynamic and environmental factors [42,51]. In long-term swimming events, especially in cold water, are similar patterns described [42,51]. Elevated proportions of Th17 cells suggest a possible immunological contribution to airway inflammation, although the causal relationship has not been conclusively established [37].

Cardiac responses showed a transient decrease in systolic performance and β-adrenergic responsiveness, consistent with exercise-induced cardiac fatigue [43–45,52,119]. A preserved cardiac output and normalization of troponin levels within 48 hours suggest functional, reversible changes rather than damage [44,52]. Possible mechanisms include downregulation of β-adrenoceptors, altered Ca² ⁺ homeostasis, and metabolic stress [44,45]. Compared to full-distance competitions, the extent of cardiac dysfunction appears to be lower, which is consistent with the reduced overall workload [44,45,52]. The incidence of cardiovascular death is low, estimated at ~1 per 50,000 triathletes [39,120]. Although experienced endurance triathletes often present with benign ECG variants, the long-term prognostic significance of acute biomarker elevations remains uncertain, reinforcing the value of individualized risk stratification [120–122].

### Implications across different distances

Across all areas, the IRONMAN® 70.3 shows consistently weaker responses compared to the full-distance triathlon, which supports the dose-response model of physiology:

- Metabolism: Fat utilization present in both races, but lower cumulative deficit. preserved muscle mass
- Muscle damage: Elevated markers in both races, but lower amplitude
- Cardiac stress: Transient in both races, but less pronounced dysfunction
- Recovery: Generally faster normalization (≤ 48 h)
- Lower dehydration, electrolyte imbalance, and cardiac stress

### Interpretation

- The IRONMAN® 70.3 appears to be physiologically tolerable for trained individuals
- Individual sodium and hydration strategies are advisable
- Targeted monitoring of compromised physiological systems is recommended for risk individuals
- Inflammatory responses should be viewed as adaptive signals, and a rest period should be planned following the race.

### Medical considerations and injury risk

Medical issues in IRONMAN® 70.3 arise from the combined effects of environmental load, prolonged exertion and segment-specific mechanical demands [39,53]. Overall risk remains low, but clinically relevant events do occur. Rare but severe events, like cardiovascular incidents, drowning, thrombotic events, and SIPE highlight the importance of individual risk assessment and environmental conditions [39,51,123].

Most medical encounters cluster in the final third of the race, informing strategic deployment of medical personnel and resources [39,53]. This pattern reflects cumulative fatigue, dehydration, and thermal strain. Musculoskeletal complaints are more common during running, suggesting the importance of eccentric loading [54]. Gastrointestinal symptoms are common among endurance athletes; this is attributed to reduced blood flow to the internal organs during prolonged physical exertion [54,124].

Organizational factors also play a crucial role in the safety of triathletes. The targeted deployment of medical resources is particularly beneficial during the running segment [39,53]. Evidence from structured race-medical systems demonstrates that proactive, segment-specific planning reduces complication severity and improves triathlete outcomes [39,53,60]. Environmental factors such as heat and UV radiation also act as additional risk factors for accidents [64]. Ventilatory efficiency and body mass acting as key determinants of recovery [62].

Compared with full-distance IRONMAN®, IRONMAN® 70.3 showed fewer injuries, shorter treatment times, and lower complication severity [39,53,60]. Whereas, in Olympic-distance competitions, a lower incidence and milder disease progression have been reported, which is consistent with the shorter duration of exposure [53,60].

### Implications across different distances

- Full-distance IRONMAN® shows higher incidence and severity of complications, and the Olympic-distance shows lower overall complication rates.
- Longer recovery and increased cumulative load the longer the race duration.

### Interpretation

- Complications are primarily due to cumulative fatigue and dehydration, not to individual events
- Most medical issues are temporary and reversible, which demonstrates the overall safety of the sport for trained athletes
- Serious incidents are rare and are associated with individual risk factors and environmental influences
- There is a correlation between the duration of the competition and the severity of complications
- Organizational strategies and medical planning reduce medical risk.
- Behavioral factors (e.g., high training volume) can indirectly increase risk

### Performance determinants and environmental modifiers

Performance in IRONMAN® 70.3 reflects the interplay among physiological capacity, tactical pacing, environmental conditions and technical execution. Cycling is consistently the strongest predictor of overall race time due to its mechanical demands, aerodynamics and the downstream consequences for running performance [24,27,32,69,125–127]. This aligns with its contribution of 50–60% to total race duration [1,82]. Pacing analyses show mid-race deceleration across performance levels, suggesting that conservative intensity regulation may mitigate premature fatigue and preserve neuromuscular output for the run segment [7,27,69]. Swimming showed weaker associations with overall performance and its predictive value diminished at higher competitive levels, indicating a ceiling effect among professionals [70,73,81]. These findings are also supported by the general literature on endurance sports and are further corroborated by the observation that accumulated competitive experience, as reflected in personal best times, is a strong predictor of performance in shorter triathlon formats [8]. However, the applicability of this relationship to middle-distance races likely depends on additional factors such as environmental stressors and technical execution.

Technical and biomechanical factors are modifiable contributors [65,67,75,128]. Distinct neuromuscular activation patterns between road and time-trial bicycle configurations influence both cycling efficiency and subsequent running performance [67,128]. Strength training has also been associated with improved outcomes [65,75,129]. Transition efficiency, which differs by sex and competitive level, further contributes to outcome variance, particularly among high-level triathletes [71,63].

Environmental load shapes race dynamics profoundly. Heat, humidity, and altitude consistently impair performance, while cooler temperatures and prior heat acclimation attenuate physiological strain [19,22]. Acclimatization to the heat mitigated this disadvantage somewhat [79], underscoring the importance of pre-competition preparation. Other factors, including sleep disturbances following travel and fluid-electrolyte changes under thermal stress, further reduce performance [48,49]. These findings underscore the need to incorporate environmental factors into strategies for pacing, preparation, and career planning.

Race characteristics and organizational context also contribute meaningfully. Course topography, elevation gain, wind exposure and competitive density influence mechanical load, pacing strategy and group dynamics even in non-drafting events [67,68,84,130]. Social-organizational factors, including event structure, crowd density and urban versus rural settings, also modulate engagement and pacing behavior rather than direct performance [50,80]. Together, these findings support a performance model in which success depends on aligning physiological capacity and technical execution with environmental and contextual demands.

Comparisons with other triathlon formats reveal both common and distance-specific influencing factors. In a review study, swimming pace remained largely stable across all race distances and predicted overall performance in amateur triathletes, but not in professional triathletes, suggesting that its predictive value decreases at higher performance levels [70,81]. In the full-distance IRONMAN®, as in the IRONMAN® 70.3, cycling also dominates, with the relative importance of pace management and energy management increasing as the duration of the race increases [70,81]. At the Olympic distance, personal best times were identified as the best performance predictor [8], underscoring the importance of accumulated competition experience. This is also evident in the fact that the fastest performance among women was achieved at the 2024 Olympic Games [9], whereas no single fastest race could be identified among men [8,9]. In the full-distance IRONMAN®, the best performances were recorded in Copenhagen, Hawaii, and Barcelona [7]. These differences highlight the need to interpret performance in the IRONMAN® 70.3 within its specific physiological and tactical context.

### Implications across different distances

- Cycling remains a key factor in both IRONMAN® 70.3 and full-distance races, but has a relatively greater influence in middle-distance races due to its higher percentage of the total race distance
- Fatigue is more pronounced during the run at longer distances, and pacing strategy becomes increasingly critical
- The predictability of overall performance based on swim time decreases as performance level and distance increase. For elite triathletes, it is a minimal distinguishing factor.
- Environmental influences are comparable across all formats, but their cumulative effect is greater in longer races.
- In Olympic-distance races, speed, tactics, and experience take precedence over energy conservation.

### Interpretation

- Performance is best explained by a trimodal model that integrates physiology, technique, and environment.
- Cycling sets the performance limit, while running under fatigue conditions determines the final result.
- Pace strategy is a primary, modifiable factor, particularly for non-elite triathletes.
- Environmental stressors act as performance inhibitors and require individual adaptation strategies.
- Technical marginal gains, such as transition times, become decisive at higher competitive levels.
- The current evidence base is robust in terms of large-scale observational data but limited in its mechanistic depth.

### Nutritional strategies

The studies found suggest nutritional strategies in IRONMAN® 70.3 are primarly determined by carbohydrate availability, hydration, and electrolyte balance are central to sustaining race intensity and supporting stable pacing across segments [85–89]. Higher carbohydrate intake consistently enhances performance and is well tolerated at intakes exceeding those commonly reported in full-distance events [85–89,131]. The absence of gastrointestinal symptoms suggests that nutritional strategies can be implemented more variably in IRONMAN® 70.3, probably due to the shorter duration and lower cumulative physiological stress [89,132].

Individual hydration and sodium replacement remains critical for maintaining hydration and plasma osmolality, particularly under heat stress [64,115]. The marked decrease in blood amino acid concentration during competition underscores the importance of adequate daily protein intake and timely recovery after training [23]. The decrease in blood amino acid concentration during competition shows no correlation with fatigue during the race. This suggests that amino acid availability is not a primary limiting factor for acute performance [23,91]. Instead, the focus should be on adequate daily protein intake and recovery after training [90,107]. This results are consistent with more general endurance recommendations highlighted targeted intake of sodium, carbohydrates, water, fat, nitrates, caffeine and protein, including 1.2–2.0 g/kg/day protein, although these guidelines have not been separately validated in the context of IRONMAN® 70.3 [90,107].

Nutritional patterns may also influence RED-S risk in female triathletes, underscoring sex-specific considerations in fueling strategy [133,134]. The association between higher fat intake and a lower incidence of a relative energy deficit during training (RED-S) in female triathletes demonstrates that total energy availability, and not just macronutrient composition, is crucial for a good outcome [92,133]. This underscores the need for individualized and gender-specific nutritional planning. Creatine supplementation shows mixed evidence, with isolated studies reporting reduced immune perturbation but no consistent performance benefit, as supported by both primary studies and reviews [93,107,135,136] The risk of supplement addiction emphasizes the need for informed triathlete education and quality-assured products [94].

### Implications across different distances

- Higher reliance on energy intake during the race, which increases with race duration
- Hydration and sodium importance increases with duration and environmental stress
- Low-carbohydrate strategies appear to be particularly disadvantageous in middle- and long-distance formats
- Supplement evidence (e.g., creatine) remains weak across endurance disciplines

### Interpretation

- Carbohydrate availability is the most important nutritional determinant of performance
- Fluid and sodium balance are critical under heat and high sweat loss
- Protein is mainly relevant for muscle recovery, not acute performance
- Amino acid depletion reflects metabolic demand rather than actual performance limitation
- Supplementation provides limited benefit and carries potential risks
- Individualized adjustments are essential due to variability in physiology and race conditions

### Age, sex and discipline-specific factors

The studies demonstrate age, sex, and discipline-specific demands interact with performance trajectories in IRONMAN® 70.3 align with established age- and sex-specific endurance patterns. Peak performance occurs in IRONMAN® 70.3 typically between 18–39 years in men and 25–39 years in women, and exhibiting narrower sex differences with advancing age [3–5,75,95]. Across triathlon formats, peak age increases with race duration from ~26 years in Olympic-distance triathlon to ~34 years in full IRONMAN® and ≥35–40 years in ultra-endurance events, reflecting the rising contribution of experience, pacing expertise and long-term aerobic development [3–5,75,95,137–139,140].

Discipline-specific age reveals further differences. For example, in the full-distance IRONMAN, the best performance in swimming is around 29 years, followed by cycling at around 31 years and running at around 35 years [139]. These results suggest an increasing importance of metabolic efficiency and fatigue resistance in disciplines with later peak performance [139]. In the IRONMAN® 70.3, this is reflected in the dominant role of cycling and the accumulated fatigue in the running segment, thus reinforcing the interaction between the order of the disciplines and the performance result. Across all formats, running shows the steepest age-related decline in performance, while cycling performance remains relatively constant [4,5].

Age-related performance decline is driven largely by reductions in V̇O₂max around 1% per year after age 30 and progressive neuromuscular remodeling, with V̇O₂max decreasing progressively after early adulthood [50,141–146]. A cause of age-related performance decline shows progressive muscle loss from the age of 50–60, which can be partially mitigated by hypertrophy-oriented training [95,147]. Women, however, show earlier muscle loss in perimenopause, which can be reduced by hormone replacement therapy [147]. The observed delayed decline in performance with age between amateur and elite athletes suggests that the losses can probably be mitigated by differences in training history and physiological reserve [3,95]. The decline in running compared to cycling supports the hypothesis that weight-bearing and neuromuscular demands accelerate performance deterioration [4,5] although in general findings high-intensity training remains effective into older adulthood [84,98,148].

Sex-specific differences in performance persist across all age groups but narrowing with advancing age, suggesting differential age trajectories between woman and men [3–5,96,97]. Across triathlon races, sex gaps range from 12–18%, largest in running (17.1%) and smallest in cycling (13.4%), while swimming typically shows the smallest absolute differences [72,149]. These differences are generally explained by physiological factors, including different muscle phenotype, higher fat mass and, lower V̇O₂max, alongside sex-specific endocrine influences [6,150–152]. However, increased fat burning and delayed glycogen depletion can mitigate these differences during longer competitions and have also shown positive effects in the swimming segment [6,11,12,153]. In opposite men show greater testosterone driven erythropoiesis and muscle cross-sectional area [6,11,12]. Discipline-specific characteristics can modulate gender differences, with mechanical and metabolic demands playing a role [63,96,149].

Training characteristics show both shared and IRONMAN® 70.3 specific features. High-volume, low-intensity training, supplemented by high-intensity sessions (polarized/pyramidal models), is a general endurance training principle that has been observed across all triathlon distances [154–157]. Structured preparation for IRONMAN® 70.3 typically spans 20–24 weeks with moderate weekly training volumes (6.5–11.5 h wk^-1^) for recreational athletes, in contrast from higher training loads typically required for full-distance events [84,98,154,158]. Increasing weekly training volume beyond 14 hours/week did not necessarily improve performance in a full IRONMAN®, suggesting a diminishing benefit at higher training loads [154,158]. Regardless of the format, training volume increases with race distance, but the optimal intensity distribution remains unclear [81,155–157]. An important difference is that cycling training proves to be the most important performance factor in IRONMAN® 70.3, both in training and in competition, and explains a large part of the performance variance, whereas the relative importance of the disciplines varies depending on the distance (e.g., swimming in the Olympic distance, running in the full IRONMAN®) [10,84,98,99].

In addition to physiological and training-related variables, multifactorial influencing factors such as experience, recovery, and psychological characteristics affect all endurance sports, regardless of the race distance [81,99,100,159–161]. Early sport participation and pain-resilience conditioning are additionally associated with improved long-term outcomes, indicating the importance of developmental factors [81,159,160].

Olympic-distance triathlon emphasizes speed and tactical execution, with an earlier peak performance (26 years) and a greater influence of swimming performance [10,95]. IRONMAN® 70.3 represents a transitional format where cycling is the dominant performance factor and pacing is important, whereas full-distance IRONMAN® places a greater emphasis on running endurance and energy management [10,139]. Ultra-endurance formats demonstrate that performance is increasingly determined by experience and fatigue resistance rather than maximum physiological capacity [95,137,138].

Additional contextual factors, such as regional differences in the athlete population, can also influence performance characteristics. For example, the age distribution among top runners varies by origin, with Asian and African athletes achieving peak performance at a younger age compared to European athletes [162]. Although these results were not specifically investigated for triathlon, they suggest that demographic and training culture factors may contribute to the observed performance patterns.

However, in IRONMAN® 70.3, the interaction of these factors with race tactics is particularly important, as the mean duration and intensity require both metabolic efficiency and tactical control. From a practical perspective, these results show that endurance principles such as aerobic development, structured training, and recovery must be adapted to the specific demands of the IRONMAN® 70.3. The focus should be on cycling efficiency, pacing strategy, and the integration of discipline-specific fatigue management.

### Implications across different distances

- The age of top triathletes increases with race duration, from a peak of around 26 years (Olympic distance) to approximately 34 years (IRONMAN®) to 35–40 years (ultra-endurance).
- Experience and race pace become increasingly important with longer race durations.
- The importance of the disciplines for overall performance shifts: swimming (Olympic distance), cycling (IRONMAN® 70.3), and running (IRONMAN®).
- The gender differences are greatest in running and smallest in swimming.
- Training volume increases with race distance, while the optimal intensity distribution remains unclear.

### Interpretation

- Cycling is considered the most stable performance factor across all age groups.
- Running contributes most to the general age-related decline in performance.
- Gender differences are primarily physiological, but are partially mitigated in longer competitions.
- Besides physiology, holistic factors such as experience, psychology, and recovery are crucial.

## Conclusion

This review demonstrates that performance in IRONMAN® 70.3 is shaped by the integrated effects of physiological capacity, pacing behavior, training structure, nutritional strategy and environmental load. Cycling consistently represents the strongest contributor to overall race outcome, emphasizing the relevance of power development, aerodynamic efficiency and disciplined intensity regulation. Age- and sex-specific performance patterns reflect established physiological trajectories, while accumulated experience allows many triathletes to sustain competitive capabilities despite progressive declines in maximal aerobic function.

Acute multisystem responses, including transient perturbations in immune activity, electrolyte balance and muscle-damage biomarkers, appear self-limiting and resolve without evidence of lasting dysfunction in trained triathletes. Nutritional strategies centered on carbohydrate availability and individualized hydration offer the clearest practical performance benefits, although discipline-specific evidence for the IRONMAN® 70.3 format remains limited. Environmental heat, humidity and altitude impose meaningful performance constraints, highlighting the need for structured acclimation and context-aware pacing. Overall, performance in IRONMAN® 70.3 reflects a dynamic interplay among physiological, environmental, technical and psychological factors. To advance evidence-based practice, future research should prioritize adequately powered, sex-balanced and age-diverse cohorts, longitudinal monitoring of training load and recovery, standardized environmental profiling and controlled interventions targeting heat adaptation, nutrition and biomechanical optimization.

### Key message

**Table:** 

Key Points	Summary
Cycling is the strongest predictor of overall IRONMAN® 70.3 performance.	Cycling explains the largest variance in total race time, exceeding swimming and running.
Peak performance occurs between 25–39 years, with sex differences diminishing at older ages.	Performance declines begin earlier in women, but sex gaps narrow after age 50.
IRONMAN® 70.3 induces transient physiological stress without persistent dysfunction.	Post-race immune, muscle-damage, and electrolyte changes normalize within hours to days.
Heat and humidity impair performance and increase medical burden.	Environmental stress reduces pacing capacity and increases dehydration risk.
Carbohydrate availability and individualized sodium intake are the most consistently supported nutritional strategies.	Higher carbohydrate intake improves performance; sodium supports hydration and heat tolerance.
Medical incidents accumulate in the final race segment.	Most adverse events occur in the last third of the race, informing medical planning.

## Supporting information

S1 File PRISMA 2020 checklist: Completed PRISMA checklist for this systematic review. (DOCX)

S2 File Minimal dataset: Completed dataset for this systematic review. (XLSX)
