# Supplementary material for: Performance, physiology, and determinants of success in IRONMAN® 70.3: A systematic review
Source: PLoS One. 2026 Jul 17;21(7):e0352506. doi: 10.1371/journal.pone.0352506 (PMC13379040; doi:10.1371/journal.pone.0352506)
Supplement: Attachment — Submitted filename: PONE-D-25-68187_Revision 1_Answer to Reviewers.docx [file pone.0352506.s004.docx]

Reviewer 1

**1. Please ensure that your manuscript meets PLOS ONE's style requirements, including those for file naming. The PLOS ONE style templates can be found at**

<https://journals.plos.org/plosone/s/file?id=wjVg/PLOSOne_formatting_sample_main_body.pdf> and

<https://journals.plos.org/plosone/s/file?id=ba62/PLOSOne_formatting_sample_title_authors_affiliations.pdf>

*Answer: We agree with the expert reviewer and corrected the file naming and the style.*

**2. Thank you for stating the following in the Acknowledgments Section of your manuscript:**

“Not funding.”

We note that you have provided funding information that is not currently declared in your Funding Statement. However, funding information should not appear in the Acknowledgments section or other areas of your manuscript. We will only publish funding information present in the Funding Statement section of the online submission form.

Please remove any funding-related text from the manuscript and let us know how you would like to update your Funding Statement. Currently, your Funding Statement reads as follows:

“The author(s) received no specific funding for this work.”

*Answer: We agree with the expert reviewer and removed the founding statement in the manuscript. Our funding statement is still the same.*

Please include your amended statements within your cover letter; we will change the online submission form on your behalf.

*Answer: We agree with the expert reviewer and have entered the financing in the correct place and removed on the wrong place.*

**3. Please provide a complete Data Availability Statement in the submission form, ensuring you include all necessary access information or a reason for why you are unable to make your data freely accessible. If your research concerns only data provided within your submission, please write "All data are in the manuscript and/or supporting information files" as your Data Availability Statement.**

*Answer: We agree with the expert reviewer and have wrote “ All relevant data underlying this systematic review are fully available within the manuscript and supporting information files and supplementary dataset File S2. As this study did not include a meta-analysis, pooled statistical analyses, or generation of original quantitative datasets, no additional raw datasets were created. All extracted study characteristics, outcomes, subgroup information, and quality assessments used to support the findings and conclusions are reported within Tables 1–9.*

**4. Please ensure that you refer to Figure 1 in your text as, if accepted, production will need this reference to link the reader to the figure.**

*Answer: We agree with the expert reviewer and have inserted Fig 1 (PRISMA flow diagram) in the text in a meaningful way.*

Following the screening process, a total of 86 studies were included in this review (Fig 1).

**Fig 1: PRISMA flow diagram of study selection for IRONMAN^®^ 70.3.** The Flow diagram illustrating identification, screening, eligibility, and inclusion of studies found for IRONMAN^®^ 70.3. Based on the study by Siddaway et al. [15].

5. We note you have included a table to which you do not refer in the text of your manuscript. Please ensure that you refer to Table 1 in your text; if accepted, production will need this reference to link the reader to the Table.

*Answer: We agree with the expert reviewer and have inserted Table 1 in the text in a meaningful way.*

Twenty-two studies examined physiological responses to IRONMAN^®^ 70.3, predominantly in small cohorts of trained male triathletes aged 25–39 years [18-36] (Tables 1 and 2).

**6. Please include captions for your Supporting Information files at the end of your manuscript, and update any in-text citations to match accordingly. Please see our Supporting Information guidelines for more information:** [**http://journals.plos.org/plosone/s/supporting-information**](http://journals.plos.org/plosone/s/supporting-information)**.**

*Answer: We agree with the expert reviewer and have added supporting information.*

This systematic review was conducted in accordance with PRISMA 2020 recommendations, with all methodological steps documented prospectively following an initial scoping phase. The PRISMA 2020 checklist is provided in S1 File.

**Supporting information**

S1 File. PRISMA 2020 checklist. Completed PRISMA checklist for this systematic review.

7. If the reviewer comments include a recommendation to cite specific previously published works, please review and evaluate these publications to determine whether they are relevant and should be cited. There is no requirement to cite these works unless the editor has indicated otherwise.

**Comments to the Author**

1. Is the manuscript technically sound, and do the data support the conclusions?

The manuscript must describe a technically sound piece of scientific research with data that supports the conclusions. Experiments must have been conducted rigorously, with appropriate controls, replication, and sample sizes. The conclusions must be drawn appropriately based on the data presented.

Reviewer #1: Yes

Reviewer #2: Partly

2. Has the statistical analysis been performed appropriately and rigorously?

Reviewer #1: No

Reviewer #2: N/A

3. Have the authors made all data underlying the findings in their manuscript fully available?

The [PLOS Data policy](http://track.editorialmanager.com/CL0/http:%2F%2Fwww.plosone.org%2Fstatic%2Fpolicies.action%23sharing/1/010f019cc5e569a1-3f919f83-6d45-4467-bd82-94b280b37498-000000/mt47Me4d4_mxZPsuUadfY22_GqqRkThGsdbg8LUc-wM=253) requires authors to make all data underlying the findings described in their manuscript fully available without restriction, with rare exception (please refer to the Data Availability Statement in the manuscript PDF file). The data should be provided as part of the manuscript or its supporting information, or deposited to a public repository. For example, in addition to summary statistics, the data points behind means, medians and variance measures should be available. If there are restrictions on publicly sharing data—e.g. participant privacy or use of data from a third party—those must be specified.

Reviewer #1: No

Reviewer #2: Yes

4. Is the manuscript presented in an intelligible fashion and written in standard English?

PLOS ONE does not copyedit accepted manuscripts, so the language in submitted articles must be clear, correct, and unambiguous. Any typographical or grammatical errors should be corrected at revision, so please note any specific errors here.

Reviewer #1: Yes

Reviewer #2: Yes

5. Review Comments to the Author

Please use the space provided to explain your answers to the questions above. You may also include additional comments for the author, including concerns about dual publication, research ethics, or publication ethics. (Please upload your review as an attachment if it exceeds 20,000 characters)

Reviewer #1: The manuscript presents a comprehensive and timely systematic review addressing performance, physiological responses, training characteristics, nutritional strategies, environmental influences, and medical considerations specific to IRONMAN® 70.3 events. The topic is highly relevant, and the authors successfully address a clear gap in the literature, as no previous review has focused exclusively on this race distance. Overall, the work is technically sound, well structured, and clearly aligned with the objectives stated in the introduction.
The review follows PRISMA 2020 guidelines, with a transparent and appropriate search strategy, clearly defined inclusion and exclusion criteria, and a robust study selection process. The synthesis of 84 studies is coherent and balanced, and the conclusions are well supported by the evidence presented. Importantly, the authors avoid overinterpretation and appropriately acknowledge the main limitations of the available literature, including small sample sizes, heterogeneous methodologies, male-dominated cohorts, and the limited number of interventional studies.
As expected for a systematic review, no new statistical analyses are performed, and this approach is justified given the heterogeneity of study designs and outcome measures. The authors accurately report and interpret statistical findings from the included studies, such as correlations and explained variance, and the use of established risk-of-bias assessment tools tailored to different study designs strengthens the methodological rigor. The decision not to conduct a meta-analysis is reasonable and clearly explained.
Data transparency is adequately addressed. Since this is a systematic review, no primary data are generated, and the authors clearly state that all information is derived from publicly available sources, in compliance with journal data availability requirements.
The manuscript is generally well written in clear and standard scientific English, with a logical structure and effective use of subheadings to guide the reader through a large body of information. Some sections, particularly in the Results and Discussion, are dense and could benefit from minor condensation or additional summarizing elements to enhance readability. Minor editorial issues, such as occasional long sentences or slight inconsistencies in terminology (e.g., use of “Half IRONMAN®” versus “IRONMAN® 70.3”), should be addressed to improve clarity, but these do not detract from the overall quality of the work.
In summary, this is a well-executed and informative systematic review that makes a meaningful contribution to the endurance sports literature. With minor revisions focused on clarity, consistency, and editorial refinement, the manuscript would be suitable for publication.

*Answer: We agree with the expert reviewer and we thank reviewer 1 for the positive and constructive evaluation of our manuscript. We have revised the manuscript with:*

Structured key elements in the discussion in a more concise and itemized manner to enhance readability.

Shortened long sentences where identified and appropriate.

Standardized terminology throughout the manuscript with consistent use of “IRONMAN® 70.3”.

Reviewer #2: Thank you for the opportunity to review the article entitled “Performance, Physiology, and Determinants of Success in IRONMAN 70.3: A Systematic Review”.
I find the article's objective to be of interest to the scientific community, as it is the first review paper on this specific triathlon distance. However, several important critical issues require significant effort on the part of the authors before publication can be considered. The main critical issues identified are: 1) lack of appropriate terminology for searching articles (the distance under consideration is commonly referred to as “middle-distance triathlon”, “70.3 IRONMAN” is the branded distance and therefore may only partially include articles published on the topic); **2) the “results” and “discussion” sections require extensive reworking, in particular, the results section contains too many sub-paragraphs presenting information in a non-systematic manner (different in terms of organisation within and between paragraphs) and often lacking information (results data, methodology), and often containing information not relevant to the subject of the study, while the discussion section is simply a discursive summary of the results section without the presence of numerical data and citations;** 3) the structure of the tables needs to be re-evaluated to allow readers to see the information more clearly and correctly; 4) the “introduction” section already presents a summary of all results of the paper, leading the reader to wonder what is the novelty and usefulness of conducting this review.
I am also leaving a series of detailed comments, which are not exhaustive, in light of the critical issues that have emerged and been reported. I hope that my comments will help the authors to improve the quality of their work.

*Answer: We agree with the expert reviewer and we thank reviewer 2 for the detailed and constructive feedback. The detailed comments were very helpful and contributed to improving the overall quality of our manuscript.*

1) Search terminology: We tested the use of the term “middle distance triathlon”, but this approach resulted in a large number of irrelevant records. Therefore, we did not pursue it further. The term “IRONMAN 70.3” yielded the most relevant results for our research question. However, we repeated the search including the missing term and, by applying the hyphenated version “middle-distance triathlon”, we were able to identify two additional relevant studies, which have now been included.

2) Results and Discussion: We revised the results section by removing multiple subsections and restructuring it in a more systematic way. The studies are now consistently presented based on reported outcomes, sample size, methodology, and key results. In the discussion section, we added appropriate references and embedded the findings into a clearer scientific context.

3) Tables: We revised all tables and added key information including sex of participants, publication year, and authors to improve clarity and completeness.

4) Introduction: We revised the introduction and placed a stronger focus on the rationale and added value of this systematic review, reducing overlap with the results section.

Line 73 and 74 – the range of peak performance seems to be large…could you be more precise?

*Answer: we agree with the expert reviewer and have narrowed down the numbers for the professional athletes.*

**Results**: Participation has increased across age groups, with pronounced growth among female and masters’ athletes. Peak performance in professional male athletes is reached at age 28, and in female athletes at age 32.

Line 81 - What do you mean by “medical incidents”? Can you give some more information? And by ‘final race segment”, do you mean the running leg?

*Answer: We agree with the expert reviewer and have clarified the meaning and the terminology.*

Environmental heat and humidity impaired performance. Most medical incidents refers to acute events including hyperthermia, dehydration, hyponatremia, gastrointestinal distress, and musculoskeletal injuries occurred in the running leg.

Line 82 - In the results section of the abstract, you do not mention any information regarding the studies analysed in relation to the total number of participants, their specifications and the type of experimental design etc. It is therefore difficult to judge them. Please insert these information in the results section or modify this section.

*Answer: We agree with the expert reviewer and have revised the abstract accordingly. We added information on the total number of participants, sample characteristics, and study design to improve the assessment of the included studies.*

Line 116 – in fact, the mentioned distance is recognised as “middle-distance triathlon”, brended as 70.3 Ironman or half-Ironman by the Ironman company. Have you chosen to analyse only events organised by the Ironman brand? Why did you make this choice?

*Answer: We agree with the expert reviewer and added “middle-distance triathlon”, at least we added 2 articles. The reason we excluded this search term was that the spelling without a hyphen generated a substantial number of irrelevant results.*

Introduction section - All the information contained in this section, which should serve as a basic introduction to the topic addressed in the paper, has been presented in the results section. If the information gathered during this review is already known and established, what is the novelty and usefulness of conducting this review?

*Answer: We thank the reviewer for this comment. The introduction was revised to focus solely on the general context and rationale for the review, removing any information that belongs exclusively to the results section. Regarding novelty: while some findings on IRONMAN® 70.3 exist in isolation, no prior systematic review has synthesized evidence across all relevant domains exclusively for this race distance. The present review therefore provides the first comprehensive, multidisciplinary synthesis specific to IRONMAN® 70.3, offering a structured evidence base that was previously unavailable to athletes, coaches, clinicians, and researchers.*

Line 159 – May you provide further information about the “increasing scientific interest” in middle-distance triathlon/ 70.3 IRONMAN?

*Answer: We agree with the expert reviewer and adjusted the context and highlighted the correlation with the increasing number of participants.*

Although the increasing number of participants suggests a greater need for specific information on the IRONMAN^®^ 70.3 research on this topic remains fragmented, with small, predominantly male samples, heterogeneous methodologies and inconsistently reported environmental and physiological metrics.

Line 177 – As stated before, the distance on which you have decided to focus your research topic is (also) known as “middle-distance triathlon”. Therefore, my question is whether the authors deliberately decided to focus solely on branded competitions (and what the reason for this choice is). Otherwise, if the the authors intended to understand the determinants of success for the distance (even non-brendized), it would be desirable to include terms that would allow for possible publications that have analysed information from non-brendized competitions and therefore recognised under the name “middle-distance triathlon”.

*Answer: We agree with the expert reviewer and added “middle-distance triathlon”.*

Line 194 – please provide the first letters of the reviewers who performed the selection process.

*Answer: We agree with the expert reviewer and added the first letters of the reviewers.*

After removal of duplicates, 86 studies met the inclusion criteria. Title and abstract screening were performed independently by three reviewers IvK, BK and KW, followed by full-text assessment when eligibility remained unclear.

Line 196 - What does “Any discrepancies were resolved through discussion until consensus was reached” stand for? Usually, a third selector is involved to resolve discrepancies between the first two selectors. It would also be useful to inform readers about the agreement between the selectors during the selection process.

*Answer: We thank the expert for their careful examination and have corrected the incorrect number. We have reached a consensus regarding uncertain texts.*

After removal of duplicates, 86 studies met the inclusion criteria. Title and abstract screening were performed independently by three reviewers IvK, BK and KW, followed by full-text assessment when eligibility remained unclear. Any discrepancies were resolved through discussion until consensus was reached.

Line 213 - Usually, the section on limitations and future research prospects is included at the end of the ‘discussion’ section. Why did the authors include this section within the methods? Personally speaking, on first reading the paper, I find it difficult to confirm and verify the statements made in this section without having yet addressed the actual results of the work carried out.

*Answer: We agree with the expert reviewer and briefly drew attention to the limitation in the methodology section to promote critical readability, and further elaborated on the limitation in the discussion section.*

**“results” - In general, the entire ‘results’ section needs to be thoroughly revised. The main critical issues I find are: the presence of numerous paragraphs that could be combined (e.g., typical complications during a race and segment-specific pattern and medical support, or age of peak performance and discipline specific peak performance); incomplete paragraphs on parameters of interest (e.g. physiological adaptations) and, in general, a lack of context for each piece of data reported (e.g. triathletes/athletes studied, competition, experimental design, etc.) that would be useful for better understanding the information provided; inconsistency in the structure of each paragraph (e.g. in some, the number of studies analysing the topic is included, while in others this information is not provided); lack of connection between individual sentences and topics addressed within a single paragraph; inclusion of data relating to other triathlon distances that are not the stated purpose of the study; many paragraphs lack clarity regarding their actual objective (e.i. environmental and race planning, race vanues); the order of the topic (inside the paragraph and between paragraphs) seems to be sometimes random. For the sake of completeness, I will leave some specific comments below, but they are certainly not exhaustive of all the issues found in the section.**

*Answer: We agree with the expert reviewer and have thoroughly revised the entire results. 1) We merged related paragraphs, for example, race complications and medical support. 2) Restructured all sections in a consistent way, reporting number of studies, sample characteristics, methodology, and key findings. 3) Added missing contextual information such as athlete population, competition type, and study design. 4) Removed or clearly marked information from other triathlon distances when not directly relevant. 5) improved the logical flow within and between paragraphs and clarified the objective of each section.*

Line 260-262 - I suggest presenting the topics addressed and explored in the same order in which they are subsequently addressed in the results section (and subsequent sections).

*Answer: We agree with the expert reviewer and ordered the topics*

The results are organized in descending order by number of studies found into physiological adaptations, competition-related complications and medical strategies, performance determinants and competition characteristics, nutritional strategies, age and gender in peak performance, as well as training characteristics and performance predictors.

Line 265 - This section does not report any results obtained from the studies cited. Please provide more information about the physiological adaptations resulting from the published studies, or refer to a table where this information can be viewed.

*Answer: We agree with the expert reviewer and added the Table 1.*

Twenty-two studies examined physiological responses to IRONMAN^®^ 70.3, predominantly in small cohorts of trained male triathletes aged 25–39 years [18-36] (Tables 1 and 2).

Line 277 - How much water was lost?

*Answer: We agree with the expert reviewer and added the water lost.*

Professional triathletes exhibited higher total body water (63.7% vs. 61%) and intracellular water, compared with recreational athletes [31]. The fluid losses during the race were comparable, 0.98l for professional athletes and 0.75l for recreational athletes, without association with overall race time [31].

Line 273 - Does this information relate only to male athletes or to female athletes as well? If so, would you be able to highlight any differences between the two sexes?

*Answer: We added the missing point.*

Across eight studies, body composition, hydration and electrolyte responses were measured (male-only cohort, n = 6-42) [5, 6,13-15,77,79,80].

Line 287 - In the studies analysed, did the athletes follow a supplementation regimen, or were these results obtained without supplementation? If so, could you provide more information about the supplementation plan that

*Answer: We agree with the expert reviewer and added the missing point. The studies do not provide any more detailed information about specific supplementation.*

Electrolyte responses were found under ad libitum intake conditions. It reported modest declines in key electrolytes including sodium declined by 3.38 mmol/L (141.72 to 138.37 mmol/L) [22].

Line 296 - As already mentioned in other sections, could you include information about whether these values are common to other endurance activities and/or within safe ranges?

*Answer: We agree with the expert reviewer and included the missing information.*

In four studies (n = 12-34 male, 4 female) markers of muscle damage documented significant increases, in creatine kinase (3- to 10-fold), myoglobin (32.8 to 654.8 mg/L) and lactate dehydrogenase (318.4 to 479 U/L; 41.71 to 191 U/L), due to the absence of pathological threshold values.

Line 305 – Please provide the total number of studies investigated on this topic (as already done in other sections)

*Answer: We agree with the expert reviewer and added the total number of studies.*

Three studies (male-only cohort, n = 6-12) documented reversible post-race proteinuria, creatinuria and hematuria [25,32]. Elevation in hepatic enzymes AST and ALT were reported but remained below harmful thresholds and normalized within 48 h [30].

Line 311 – Please change the arabic numbering of the studies to alphabetical, as done in other sections.

*Answer: We agree with the expert reviewer and changed the Arabic numbering.*

Across ten studies (n = 6-26 male, 3 female and 13-57 sex not reported), oxidative stress markers (oxygen radicals, DNA breaks) and inflammatory cytokines (IL-6, IL-10, TNF-α) increased post-race and returned to baseline within 48 h [23-26,28-30,32,34,35].

Line 315 - Here, but also throughout the paper in general, I suggest using the term ‘triathletes’ to make it clear that the information is specific to this particular population, rather than using the more generic term ‘athletes’.

*Answer: We agree with the expert reviewer and changed in the paper athletes to triathletes.*

Line 319 - As in previous comments, it is unclear whether this information was collected on ‘triathletes’ or from general studies on ‘athletes’. Also, include the number of studies that have investigated this topic.

*Answer: We agree with the expert reviewer and changed in the paper athletes to triathletes where necessary, as well as we included the number of studies.*

Four studies documented respiratory conditions in triathletes, with documented cases of asthma, airway hyperreactivity and swimming-induced pulmonary edema (SIPE) [38-41].

Line 334 – Because the review is focused on 70.3, I suggest eliminate this information about full-distance.

*Answer: We agree with the expert reviewer and eliminated the sentence about different competitions.*

Line 339 – Evaluate the use of the term “racing” and homologate within the whole text

*Answer: We agree with the expert reviewer and homologated race and competition in the whole text.*

Line 340 – please, provide information about the occurence of each mentioned issue.

*Answer: We agree with the expert reviewer and have added provide information.*

The most common issues in one measured race were hyperthermia and dehydration [36] and in an other race were mainly 27.6% gastrointestinal complaints, 25.4% musculoskeletal injury and myalgia [36].

Line 337 and 343 – I suggest merging these sections. Evaluate to merge also the “medical support” section with them.

*Answer: We agree with the expert reviewer and have merge these sections. We kept the medical support and race-related complications together.*

Line 362 – Please provide more information about the severity of the sun exposure presented, compared with guidelines on the topic.

*Answer: We agree with the expert reviewer and added the severity of the sun exposure.*

Sun exposure reached 1.2 SED/h during cycling and 3.0 SED/h during running, exceeding the minimum erythema dose of approximately 1.0 SED for fair-skinned individuals up to 5.0 SED for dark-skinned individuals [59].

Line 364-365 - The first part of this statement has already been provided in the previous sections. I suggest inserting this statement (in full) only once.

*Answer: We agree with the expert reviewer and have removed the sentence.*

Line 368-369 - Is this information specific to the subject in question or general?!

*Answer: We agree with the expert reviewer and have clarified the information.*

Structured on-course medical systems reduced treatment times and improved outcomes in IRONMAN^®^ 70.3 [47,51].

Line 370-372 - In its current form, this sentence seems rather disconnected from the rest and provides very little information. Please elaborate further and consider where to insert this information (assuming it is actually relevant to the topic).

*Answer: We agree with the expert reviewer and have connected this sentence. We mentioned it to show that, apart from this study, no other studies on mental health exist in this discipline.*

Most studies focused on physiological aspects, however one psychological study reported increased maladaptive or addictive training behavior in individuals training more than 10 h·week⁻¹ and in triathletes competing in longer events [60].

Line 376 – Please check the term “environmental” used.

*Answer: We agree with the expert reviewer and have removed the term.*

**Performance determinants and race characteristics**

Line 378 – The review is focused on just 70.3. I suggest only including information relating to the subject under analysis and not including information relating to other subjects, unless for the purpose of making comparisons where necessary (which should be carried out in the ‘discussion’ section rather than in the ‘results’)

*Answer: We agree with the expert reviewer and have removed the full distance triathlon.*

Across IRONMAN^®^ 70.3, cycling consistently emerged as the strongest predictor of overall performance [10,13,14,65,66,70], accounting for 50–60% of race duration [1,72], correlations with finishing time reached 0.85.

Line 374 – In fact, this section lacks information about environmental determinants on 70.3. please, change the title or provide further information

*Answer: We agree with the expert reviewer and have removed environmental.*

**Performance determinants and race characteristics**

“performance strategies” - This section contains various pieces of information, but without going into sufficient detail and seemingly disconnected from one sentence to the next. I suggest revising the entire section, trying to provide more details and find a connection between the various results.

*Answer: We agree with the expert reviewer and have connected the section with integrate details.*

Performance strategies were examined in relation to race execution, pacing, and biomechanics. Analyses (n = 858 male, 380 female) revealed speed declines of 1.14 km/h in the second half of cycling and 0.37 km/h (professionals) to 0.6 km/h (amateurs) in running, with larger decrements observed in men, middle-aged, and lower-performing triathletes [62]. A conservative pacing strategy with a glycogen preservation were detected as particularly important for maintaining running performance in the final race segment [65].

Biomechanical and technical factors further modulate performance outcomes. In a small cohort (n = 3 male, 2 female), the TRI position was associated with optimized force application during cycling [63]. Strength training has been shown to produce better results (male-only cohort, n = 16) [37,73]. Transition efficiency, which improved among top-10 finishers over time, although sex differences in transition times persist across age groups [68,74]. Swim pacing remained relatively stable across race distances and was associated with overall performance in amateur but not professional triathletes [64,67].

“Race venues” - If this section aims to highlight the competitions in which athletes have achieved the best results (and therefore are favourable for obtaining the best absolute times), I suggest changing the title of the paragraph. My question, in addition to wondering whether this information is actually useful, is whether only published scientific articles were taken into account when compiling this section, or whether the data was verified directly in the results databases?
Furthermore, as mentioned above, including information on other triathlon distances seems to me to be beyond the stated purpose of the review and misleading for the reader, so I would remove these information.

*Answer: We agree with the expert reviewer and have been revised the title accordingly. Information on locations associated with the best performances may be relevant for career planning, as it could facilitate qualification. Differentiation from other competitions is considered helpful for the interpretation and evaluation of the data, we removed them to the discussion section. We have only used published scientific articles.*

**Peak performance and participation trends**

“enviromental stressors” - It is unclear whether the results reported in this section are all related to triathletes or general. Please clarify.

*Answer: We agree with the expert reviewer and have clarified the section.*

Environmental load exerted a measurable influence on performance in IRONMAN^®^ 70.3, with speed declined at temperatures exceeding 25 °C, humidity above 70%, and at altitude [76,77].

Line 420-554 - I found it really difficult to continue with a detailed review of the individual points, which are extremely similar to what has already been mentioned in the previous comments. A thorough review of the entire results section is highly recommended.

*Answer: We agree with the expert reviewer and have reviewed the text again. For clarity’s sake, it should be noted here that the comparisons, where found, to the next shorter and next longer triathlon distances are intended to help distinguish between them. As mentioned at the beginning, many athletes progress to longer triathlon distances gradually. We removed the comparisons in the discussion section.*

“Discussion” - There are no citations in this section. Although all studies and results have already been presented in the previous section, references are also essential in the discussion section. Moreover, this entire section appears to be a summary of the information presented in the results section, without any numerical/quantitative references and (often) without any actual discussion. The authors may consider combining the results and discussion sections to optimise the results section.*Answer: We thank the reviewer for this important observation. The Discussion section has been substantially revised. References and numerical data have been integrated throughout each thematic sub-section. The discussion now provides mechanistic interpretation and contextualizes the IRONMAN® 70.3 findings within the broader endurance-sport literature, rather than merely summarizing the results. Each thematic block was extended with an “Implications across different distances” and an “Interpretation” sub-section to clearly separate description from interpretation.*

*Answer: We thank the expert reviewer for this important observation. We have added the missing references and substantially revised the discussion section. We appreciate the suggestion to combine the results and discussion sections. However, we chose to keep them separate to ensure a clear distinction between reported findings and their interpretation.*

Line 566-567 - As mentioned several times in previous comments, the results section includes information on distances and disciplines other than 70.3. However, the stated purpose of the study is to analyse only the 70.3 distance, and any comparison with other distances and/or disciplines should be addressed in the discussion section. Alternatively, we recommend revising the scope of the study to include a systematic collection of information on all other distances and/or disciplines of interest.

*Answer: We agree with the expert reviewer. Comparisons with other triathlon formats (full distance IRONMAN® and Olympic distance) have been relocated from the results section to the discussion, where they appear under dedicated “Implications across different distances” headings within each thematic block. The results section now reports exclusively on IRONMAN® 70.3 data.*

Line 568-574 - All the statements made here are not supported by what was shown in the previous section of the results, in which this information is not reported at all (it is only partially recognisable in the tables). Please highlight these gaps so that we can proceed with a proper discussion of the results.

*Answer: We agree with the expert reviewer. The discussion has been revised to ensure that all statements are directly grounded in data reported in the results section or the corresponding tables. Where quantitative values or citations were absent, they have now been added. Methodological limitations and evidence gaps are explicitly addressed in the dedicated limitations paragraph of the Discussion.*

Tables - The tables are difficult for readers to read and understand. The first column, called ‘reference numbers’, seems to show the number of subjects for each study (but the distinction between male and female is unclear). The title of the study may not be such important information to include; rather, I suggest including more reference information (e.g., name of the first author and year of publication, in addition to the citation number as currently included). Finally, greater effort is needed on the part of the authors to improve the presentation of the results of the individual studies, which is extremely confusing and unclear.

*Answer: We agree with the expert reviewer and have added the distinction between male and female, have changed ‘reference numbers’ to ‘study participants’, have added the title and year.*

References - It is necessary to carefully check all references as they do not appear to be standardised in style and are lacking in information; in particular, none of them include the DOI.

*Answer: We agree with the expert reviewer and have carefully reviewed all references. DOIs have been added to all references where available. The formatting has been standardized across the entire reference list to follow a consistent citation style.*

**Do you want your identity to be public for this peer review?** For information about this choice, including consent withdrawal, please see our [Privacy Policy](https://track.editorialmanager.com/CL0/https:%2F%2Fwww.plos.org%2Fprivacy-policy/1/010f019cc5e569a1-3f919f83-6d45-4467-bd82-94b280b37498-000000/p6VGgw87NYUCeyPrt5pqT_v7CZUxIMT04BLAGQQlu4E=253).

Reviewer #1: **Yes:** Álvaro Miguel-Ortega

*Answer: We appreciate the trust you have placed in us.*

Reviewer #2: No

*Answer: We respect your decision and thank you for your help in improving the manuscript.*
