# Supplementary material for: Performance, physiology, and determinants of success in IRONMAN® 70.3: A systematic review
Source: PLoS One. 2026 Jul 17;21(7):e0352506. doi: 10.1371/journal.pone.0352506 (PMC13379040; doi:10.1371/journal.pone.0352506)
Supplement: Peer review history

# Peer review: Decision Letter 0

**DOI:** 10.1371/journal.pone.0352506.r001

**Authors:** Stefano Amatori

6 Mar 2026

PONE-D-25-68187

Performance, Physiology, and Determinants of Success in IRONMAN® 70.3: A Systematic Review

PLOS One

Dear Dr. Knechtle,

Thank you for submitting your manuscript to PLOS ONE. After careful consideration, we feel that it has merit but does not fully meet PLOS ONE’s publication criteria as it currently stands. Therefore, we invite you to submit a revised version of the manuscript that addresses the points raised during the review process.

Please submit your revised manuscript by Apr 20 2026 11:59PM. If you will need more time than this to complete your revisions, please reply to this message or contact the journal office at plosone@plos.org. When you’re ready to submit your revision, log on to https://www.editorialmanager.com/pone/ and select the 'Submissions Needing Revision' folder to locate your manuscript file.

Please include the following items when submitting your revised manuscript:

- A letter that responds to each point raised by the academic editor and reviewer(s). You should upload this letter as a separate file labeled 'Response to Reviewers'.
- A marked-up copy of your manuscript that highlights changes made to the original version. You should upload this as a separate file labeled 'Revised Manuscript with Track Changes'.
- An unmarked version of your revised paper without tracked changes. You should upload this as a separate file labeled 'Manuscript'.

If you would like to make changes to your financial disclosure, please include your updated statement in your cover letter. Guidelines for resubmitting your figure files are available below the reviewer comments at the end of this letter.

If applicable, we recommend that you deposit your laboratory protocols in protocols.io to enhance the reproducibility of your results. Protocols.io assigns your protocol its own identifier (DOI) so that it can be cited independently in the future. For instructions see: https://journals.plos.org/plosone/s/submission-guidelines#loc-laboratory-protocols. Additionally, PLOS ONE offers an option for publishing peer-reviewed Lab Protocol articles, which describe protocols hosted on protocols.io. Read more information on sharing protocols at https://plos.org/protocols?utm_medium=editorial-email&utm_source=authorletters&utm_campaign=protocols.

We look forward to receiving your revised manuscript.

Kind regards,

Stefano Amatori, Ph.D.

Academic Editor

PLOS One

Journal Requirements:

When submitting your revision, we need you to address these additional requirements.

1. Please ensure that your manuscript meets PLOS ONE's style requirements, including those for file naming. The PLOS ONE style templates can be found at

https://journals.plos.org/plosone/s/file?id=wjVg/PLOSOne_formatting_sample_main_body.pdf and

https://journals.plos.org/plosone/s/file?id=ba62/PLOSOne_formatting_sample_title_authors_affiliations.pdf

2. Thank you for stating the following in the Acknowledgments Section of your manuscript:

“Not funding.”

We note that you have provided funding information that is not currently declared in your Funding Statement. However, funding information should not appear in the Acknowledgments section or other areas of your manuscript. We will only publish funding information present in the Funding Statement section of the online submission form.

Please remove any funding-related text from the manuscript and let us know how you would like to update your Funding Statement. Currently, your Funding Statement reads as follows:

“The author(s) received no specific funding for this work.”

Please include your amended statements within your cover letter; we will change the online submission form on your behalf.

3. Please provide a complete Data Availability Statement in the submission form, ensuring you include all necessary access information or a reason for why you are unable to make your data freely accessible. If your research concerns only data provided within your submission, please write "All data are in the manuscript and/or supporting information files" as your Data Availability Statement.

4. Please ensure that you refer to Figure 1 in your text as, if accepted, production will need this reference to link the reader to the figure.

5. We note you have included a table to which you do not refer in the text of your manuscript. Please ensure that you refer to Table 1 in your text; if accepted, production will need this reference to link the reader to the Table.

6. Please include captions for your Supporting Information files at the end of your manuscript, and update any in-text citations to match accordingly. Please see our Supporting Information guidelines for more information: http://journals.plos.org/plosone/s/supporting-information.

7. If the reviewer comments include a recommendation to cite specific previously published works, please review and evaluate these publications to determine whether they are relevant and should be cited. There is no requirement to cite these works unless the editor has indicated otherwise.

[Note: HTML markup is below. Please do not edit.]

Reviewers' comments:

Reviewer's Responses to Questions

**Comments to the Author**

1. Is the manuscript technically sound, and do the data support the conclusions?

The manuscript must describe a technically sound piece of scientific research with data that supports the conclusions. Experiments must have been conducted rigorously, with appropriate controls, replication, and sample sizes. The conclusions must be drawn appropriately based on the data presented.

Reviewer #1: Yes

Reviewer #2: Partly

**********

2. Has the statistical analysis been performed appropriately and rigorously?

Reviewer #1: No

Reviewer #2: N/A

**********

3. Have the authors made all data underlying the findings in their manuscript fully available?

The PLOS Data policy requires authors to make all data underlying the findings described in their manuscript fully available without restriction, with rare exception (please refer to the Data Availability Statement in the manuscript PDF file). The data should be provided as part of the manuscript or its supporting information, or deposited to a public repository. For example, in addition to summary statistics, the data points behind means, medians and variance measures should be available. If there are restrictions on publicly sharing data—e.g. participant privacy or use of data from a third party—those must be specified.

Reviewer #1: No

Reviewer #2: Yes

**********

4. Is the manuscript presented in an intelligible fashion and written in standard English?

PLOS ONE does not copyedit accepted manuscripts, so the language in submitted articles must be clear, correct, and unambiguous. Any typographical or grammatical errors should be corrected at revision, so please note any specific errors here.

Reviewer #1: Yes

Reviewer #2: Yes

**********

5. Review Comments to the Author

Please use the space provided to explain your answers to the questions above. You may also include additional comments for the author, including concerns about dual publication, research ethics, or publication ethics. (Please upload your review as an attachment if it exceeds 20,000 characters)

Reviewer #1: The manuscript presents a comprehensive and timely systematic review addressing performance, physiological responses, training characteristics, nutritional strategies, environmental influences, and medical considerations specific to IRONMAN® 70.3 events. The topic is highly relevant, and the authors successfully address a clear gap in the literature, as no previous review has focused exclusively on this race distance. Overall, the work is technically sound, well structured, and clearly aligned with the objectives stated in the introduction.

The review follows PRISMA 2020 guidelines, with a transparent and appropriate search strategy, clearly defined inclusion and exclusion criteria, and a robust study selection process. The synthesis of 84 studies is coherent and balanced, and the conclusions are well supported by the evidence presented. Importantly, the authors avoid overinterpretation and appropriately acknowledge the main limitations of the available literature, including small sample sizes, heterogeneous methodologies, male-dominated cohorts, and the limited number of interventional studies.

As expected for a systematic review, no new statistical analyses are performed, and this approach is justified given the heterogeneity of study designs and outcome measures. The authors accurately report and interpret statistical findings from the included studies, such as correlations and explained variance, and the use of established risk-of-bias assessment tools tailored to different study designs strengthens the methodological rigor. The decision not to conduct a meta-analysis is reasonable and clearly explained.

Data transparency is adequately addressed. Since this is a systematic review, no primary data are generated, and the authors clearly state that all information is derived from publicly available sources, in compliance with journal data availability requirements.

The manuscript is generally well written in clear and standard scientific English, with a logical structure and effective use of subheadings to guide the reader through a large body of information. Some sections, particularly in the Results and Discussion, are dense and could benefit from minor condensation or additional summarizing elements to enhance readability. Minor editorial issues, such as occasional long sentences or slight inconsistencies in terminology (e.g., use of “Half IRONMAN®” versus “IRONMAN® 70.3”), should be addressed to improve clarity, but these do not detract from the overall quality of the work.

In summary, this is a well-executed and informative systematic review that makes a meaningful contribution to the endurance sports literature. With minor revisions focused on clarity, consistency, and editorial refinement, the manuscript would be suitable for publication.

Reviewer #2: Thank you for the opportunity to review the article entitled “Performance, Physiology, and Determinants of Success in IRONMAN 70.3: A Systematic Review”.

I find the article's objective to be of interest to the scientific community, as it is the first review paper on this specific triathlon distance. However, several important critical issues require significant effort on the part of the authors before publication can be considered. The main critical issues identified are: 1) lack of appropriate terminology for searching articles (the distance under consideration is commonly referred to as “middle-distance triathlon”, “70.3 IRONMAN” is the branded distance and therefore may only partially include articles published on the topic); 2) the “results” and “discussion” sections require extensive reworking, in particular, the results section contains too many sub-paragraphs presenting information in a non-systematic manner (different in terms of organisation within and between paragraphs) and often lacking information (results data, methodology), and often containing information not relevant to the subject of the study, while the discussion section is simply a discursive summary of the results section without the presence of numerical data and citations; 3) the structure of the tables needs to be re-evaluated to allow readers to see the information more clearly and correctly; 4) the “introduction” section already presents a summary of all results of the paper, leading the reader to wonder what is the novelty and usefulness of conducting this review.

I am also leaving a series of detailed comments, which are not exhaustive, in light of the critical issues that have emerged and been reported. I hope that my comments will help the authors to improve the quality of their work.

Line 73 and 74 – the range of peak performance seems to be large…could you be more precise?

Line 81 - What do you mean by “medical incidents”? Can you give some more information? And by ‘final race segment”, do you mean the running leg?

Line 82 - In the results section of the abstract, you do not mention any information regarding the studies analysed in relation to the total number of participants, their specifications and the type of experimental design etc. It is therefore difficult to judge them. Please insert these information in the results section or modify this section.

Line 116 – in fact, the mentioned distance is recognised as “middle-distance triathlon”, brended as 70.3 Ironman or half-Ironman by the Ironman company. Have you chosen to analyse only events organised by the Ironman brand? Why did you make this choice?

Introduction section - All the information contained in this section, which should serve as a basic introduction to the topic addressed in the paper, has been presented in the results section. If the information gathered during this review is already known and established, what is the novelty and usefulness of conducting this review?

Line 159 – May you provide further information about the “increasing scientific interest” in middle-distance triathlon/ 70.3 IRONMAN?

Line 177 – As stated before, the distance on which you have decided to focus your research topic is (also) known as “middle-distance triathlon”. Therefore, my question is whether the authors deliberately decided to focus solely on branded competitions (and what the reason for this choice is). Otherwise, if the the authors intended to understand the determinants of success for the distance (even non-brendized), it would be desirable to include terms that would allow for possible publications that have analysed information from non-brendized competitions and therefore recognised under the name “middle-distance triathlon”.

Line 194 – please provide the first letters of the reviewers who performed the selection process.

Line 196 - What does “Any discrepancies were resolved through discussion until consensus was reached” stand for? Usually, a third selector is involved to resolve discrepancies between the first two selectors. It would also be useful to inform readers about the agreement between the selectors during the selection process.

Line 213 - Usually, the section on limitations and future research prospects is included at the end of the ‘discussion’ section. Why did the authors include this section within the methods? Personally speaking, on first reading the paper, I find it difficult to confirm and verify the statements made in this section without having yet addressed the actual results of the work carried out.

“results” - In general, the entire ‘results’ section needs to be thoroughly revised. The main critical issues I find are: the presence of numerous paragraphs that could be combined (e.g., typical complications during a race and segment-specific pattern and medical support, or age of peak performance and discipline specific peak performance); incomplete paragraphs on parameters of interest (e.g. physiological adaptations) and, in general, a lack of context for each piece of data reported (e.g. triathletes/athletes studied, competition, experimental design, etc.) that would be useful for better understanding the information provided; inconsistency in the structure of each paragraph (e.g. in some, the number of studies analysing the topic is included, while in others this information is not provided); lack of connection between individual sentences and topics addressed within a single paragraph; inclusion of data relating to other triathlon distances that are not the stated purpose of the study; many paragraphs lack clarity regarding their actual objective (e.i. environmental and race planning, race vanues); the order of the topic (inside the paragraph and between paragraphs) seems to be sometimes random. For the sake of completeness, I will leave some specific comments below, but they are certainly not exhaustive of all the issues found in the section.

Line 260-262 - I suggest presenting the topics addressed and explored in the same order in which they are subsequently addressed in the results section (and subsequent sections).

Line 265 - This section does not report any results obtained from the studies cited. Please provide more information about the physiological adaptations resulting from the published studies, or refer to a table where this information can be viewed.

Line 277 - How much water was lost?

Line 273 - Does this information relate only to male athletes or to female athletes as well? If so, would you be able to highlight any differences between the two sexes?

Line 287 - In the studies analysed, did the athletes follow a supplementation regimen, or were these results obtained without supplementation? If so, could you provide more information about the supplementation plan that

Line 296 - As already mentioned in other sections, could you include information about whether these values are common to other endurance activities and/or within safe ranges?

Line 305 – Please provide the total number of studies investigated on this topic (as already done in other sections)

Line 311 – Please change the arabic numbering of the studies to alphabetical, as done in other sections.

Line 315 - Here, but also throughout the paper in general, I suggest using the term ‘triathletes’ to make it clear that the information is specific to this particular population, rather than using the more generic term ‘athletes’.

Line 319 - As in previous comments, it is unclear whether this information was collected on ‘triathletes’ or from general studies on ‘athletes’. Also, include the number of studies that have investigated this topic.

Line 334 – Because the review is focused on 70.3, I suggest eliminate this information about full-distance.

Line 339 – Evaluate the use of the term “racing” and homologate within the whole text

Line 340 – please, provide information about the occurence of each mentioned issue.

Line 337 and 343 – I suggest merging these sections. Evaluate to merge also the “medical support” section with them.

Line 362 – Please provide more information about the severity of the sun exposure presented, compared with guidelines on the topic.

Line 364-365 - The first part of this statement has already been provided in the previous sections. I suggest inserting this statement (in full) only once.

Line 368-369 - Is this information specific to the subject in question or general?!

Line 370-372 - In its current form, this sentence seems rather disconnected from the rest and provides very little information. Please elaborate further and consider where to insert this information (assuming it is actually relevant to the topic).

Line 376 – Please check the term “envariomental” used.

Line 378 – The review is focused on just 70.3. I suggest only including information relating to the subject under analysis and not including information relating to other subjects, unless for the purpose of making comparisons where necessary (which should be carried out in the ‘discussion’ section rather than in the ‘results’)

Line 374 – In fact, this section lacks information about environmental determinants on 70.3. please, change the title or provide further information

“performance strategies” - This section contains various pieces of information, but without going into sufficient detail and seemingly disconnected from one sentence to the next. I suggest revising the entire section, trying to provide more details and find a connection between the various results.

“Race venues” - If this section aims to highlight the competitions in which athletes have achieved the best results (and therefore are favourable for obtaining the best absolute times), I suggest changing the title of the paragraph. My question, in addition to wondering whether this information is actually useful, is whether only published scientific articles were taken into account when compiling this section, or whether the data was verified directly in the results databases?

Furthermore, as mentioned above, including information on other triathlon distances seems to me to be beyond the stated purpose of the review and misleading for the reader, so I would remove these information.

“enviromental stressors” - It is unclear whether the results reported in this section are all related to triathletes or general. Please clarify.

Line 420-554 - I found it really difficult to continue with a detailed review of the individual points, which are extremely similar to what has already been mentioned in the previous comments. A thorough review of the entire results section is highly recommended.

“Discussion” - There are no citations in this section. Although all studies and results have already been presented in the previous section, references are also essential in the discussion section. Moreover, this entire section appears to be a summary of the information presented in the results section, without any numerical/quantitative references and (often) without any actual discussion. The authors may consider combining the results and discussion sections to optimise the results section.

Line 566-567 - As mentioned several times in previous comments, the results section includes information on distances and disciplines other than 70.3. However, the stated purpose of the study is to analyse only the 70.3 distance, and any comparison with other distances and/or disciplines should be addressed in the discussion section. Alternatively, we recommend revising the scope of the study to include a systematic collection of information on all other distances and/or disciplines of interest.

Line 568-574 - All the statements made here are not supported by what was shown in the previous section of the results, in which this information is not reported at all (it is only partially recognisable in the tables). Please highlight these gaps so that we can proceed with a proper discussion of the results.

Tables - The tables are difficult for readers to read and understand. The first column, called ‘reference numbers’, seems to show the number of subjects for each study (but the distinction between male and female is unclear). The title of the study may not be such important information to include; rather, I suggest including more reference information (e.g., name of the first author and year of publication, in addition to the citation number as currently included). Finally, greater effort is needed on the part of the authors to improve the presentation of the results of the individual studies, which is extremely confusing and unclear.

References - It is necessary to carefully check all references as they do not appear to be standardised in style and are lacking in information; in particular, none of them include the DOI.

**********

6. PLOS authors have the option to publish the peer review history of their article (what does this mean?). If published, this will include your full peer review and any attached files.

If you choose “no”, your identity will remain anonymous but your review may still be made public.

**Do you want your identity to be public for this peer review?**  For information about this choice, including consent withdrawal, please see our Privacy Policy.

Reviewer #1: **Yes:** Álvaro Miguel-Ortega

Reviewer #2: No

**********

[NOTE: If reviewer comments were submitted as an attachment file, they will be attached to this email and accessible via the submission site. Please log into your account, locate the manuscript record, and check for the action link "View Attachments". If this link does not appear, there are no attachment files.]

To ensure your figures meet our technical requirements, please review our figure guidelines: https://journals.plos.org/plosone/s/figures

You may also use PLOS’s free figure tool, NAAS, to help you prepare publication quality figures: https://journals.plos.org/plosone/s/figures#loc-tools-for-figure-preparation.

NAAS will assess whether your figures meet our technical requirements by comparing each figure against our figure specifications.

# Author comment: Author response to Decision Letter 1

**DOI:** 10.1371/journal.pone.0352506.r002

26 May 2026

Reviewer 1

1. Please ensure that your manuscript meets PLOS ONE's style requirements, including those for file naming. The PLOS ONE style templates can be found at

https://journals.plos.org/plosone/s/file?id=wjVg/PLOSOne_formatting_sample_main_body.pdf and

https://journals.plos.org/plosone/s/file?id=ba62/PLOSOne_formatting_sample_title_authors_affiliations.pdf

Answer: We agree with the expert reviewer and corrected the file naming and the style.

2. Thank you for stating the following in the Acknowledgments Section of your manuscript:

“Not funding.”

We note that you have provided funding information that is not currently declared in your Funding Statement. However, funding information should not appear in the Acknowledgments section or other areas of your manuscript. We will only publish funding information present in the Funding Statement section of the online submission form.

Please remove any funding-related text from the manuscript and let us know how you would like to update your Funding Statement. Currently, your Funding Statement reads as follows:

“The author(s) received no specific funding for this work.”

Answer: We agree with the expert reviewer and removed the founding statement in the manuscript. Our funding statement is still the same.

Please include your amended statements within your cover letter; we will change the online submission form on your behalf.

Answer: We agree with the expert reviewer and have entered the financing in the correct place and removed on the wrong place.

3. Please provide a complete Data Availability Statement in the submission form, ensuring you include all necessary access information or a reason for why you are unable to make your data freely accessible. If your research concerns only data provided within your submission, please write "All data are in the manuscript and/or supporting information files" as your Data Availability Statement.

Answer: We agree with the expert reviewer and have wrote “ All relevant data underlying this systematic review are fully available within the manuscript and supporting information files and supplementary dataset File S2. As this study did not include a meta-analysis, pooled statistical analyses, or generation of original quantitative datasets, no additional raw datasets were created. All extracted study characteristics, outcomes, subgroup information, and quality assessments used to support the findings and conclusions are reported within Tables 1–9.

4. Please ensure that you refer to Figure 1 in your text as, if accepted, production will need this reference to link the reader to the figure.

Answer: We agree with the expert reviewer and have inserted Fig 1 (PRISMA flow diagram) in the text in a meaningful way.

Following the screening process, a total of 86 studies were included in this review (Fig 1).

Fig 1: PRISMA flow diagram of study selection for IRONMAN® 70.3. The Flow diagram illustrating identification, screening, eligibility, and inclusion of studies found for IRONMAN® 70.3. Based on the study by Siddaway et al. [15].

5. We note you have included a table to which you do not refer in the text of your manuscript. Please ensure that you refer to Table 1 in your text; if accepted, production will need this reference to link the reader to the Table.

Answer: We agree with the expert reviewer and have inserted Table 1 in the text in a meaningful way.

Twenty-two studies examined physiological responses to IRONMAN® 70.3, predominantly in small cohorts of trained male triathletes aged 25–39 years [18-36] (Tables 1 and 2).

6. Please include captions for your Supporting Information files at the end of your manuscript, and update any in-text citations to match accordingly. Please see our Supporting Information guidelines for more information: http://journals.plos.org/plosone/s/supporting-information.

Answer: We agree with the expert reviewer and have added supporting information.

This systematic review was conducted in accordance with PRISMA 2020 recommendations, with all methodological steps documented prospectively following an initial scoping phase. The PRISMA 2020 checklist is provided in S1 File.

Supporting information

S1 File. PRISMA 2020 checklist. Completed PRISMA checklist for this systematic review.

7. If the reviewer comments include a recommendation to cite specific previously published works, please review and evaluate these publications to determine whether they are relevant and should be cited. There is no requirement to cite these works unless the editor has indicated otherwise.

Comments to the Author

1. Is the manuscript technically sound, and do the data support the conclusions?

The manuscript must describe a technically sound piece of scientific research with data that supports the conclusions. Experiments must have been conducted rigorously, with appropriate controls, replication, and sample sizes. The conclusions must be drawn appropriately based on the data presented.

Reviewer #1: Yes

Reviewer #2: Partly

________________________________________

2. Has the statistical analysis been performed appropriately and rigorously?

Reviewer #1: No

Reviewer #2: N/A

________________________________________

3. Have the authors made all data underlying the findings in their manuscript fully available?

The PLOS Data policy requires authors to make all data underlying the findings described in their manuscript fully available without restriction, with rare exception (please refer to the Data Availability Statement in the manuscript PDF file). The data should be provided as part of the manuscript or its supporting information, or deposited to a public repository. For example, in addition to summary statistics, the data points behind means, medians and variance measures should be available. If there are restrictions on publicly sharing data—e.g. participant privacy or use of data from a third party—those must be specified.

Reviewer #1: No

Reviewer #2: Yes

________________________________________

4. Is the manuscript presented in an intelligible fashion and written in standard English?

PLOS ONE does not copyedit accepted manuscripts, so the language in submitted articles must be clear, correct, and unambiguous. Any typographical or grammatical errors should be corrected at revision, so please note any specific errors here.

Reviewer #1: Yes

Reviewer #2: Yes

________________________________________

5. Review Comments to the Author

Please use the space provided to explain your answers to the questions above. You may also include additional comments for the author, including concerns about dual publication, research ethics, or publication ethics. (Please upload your review as an attachment if it exceeds 20,000 characters)

Reviewer #1: The manuscript presents a comprehensive and timely systematic review addressing performance, physiological responses, training characteristics, nutritional strategies, environmental influences, and medical considerations specific to IRONMAN® 70.3 events. The topic is highly relevant, and the authors successfully address a clear gap in the literature, as no previous review has focused exclusively on this race distance. Overall, the work is technically sound, well structured, and clearly aligned with the objectives stated in the introduction.

The review follows PRISMA 2020 guidelines, with a transparent and appropriate search strategy, clearly defined inclusion and exclusion criteria, and a robust study selection process. The synthesis of 84 studies is coherent and balanced, and the conclusions are well supported by the evidence presented. Importantly, the authors avoid overinterpretation and appropriately acknowledge the main limitations of the available literature, including small sample sizes, heterogeneous methodologies, male-dominated cohorts, and the limited number of interventional studies.

As expected for a systematic review, no new statistical analyses are performed, and this approach is justified given the heterogeneity of study designs and outcome measures. The authors accurately report and interpret statistical findings from the included studies, such as correlations and explained variance, and the use of established risk-of-bias assessment tools tailored to different study designs strengthens the methodological rigor. The decision not to conduct a meta-analysis is reasonable and clearly explained.

Data transparency is adequately addressed. Since this is a systematic review, no primary data are generated, and the authors clearly state that all information is derived from publicly available sources, in compliance with journal data availability requirements.

The manuscript is generally well written in clear and standard scientific English, with a logical structure and effective use of subheadings to guide the reader through a large body of information. Some sections, particularly in the Results and Discussion, are dense and could benefit from minor condensation or additional summarizing elements to enhance readability. Minor editorial issues, such as occasional long sentences or slight inconsistencies in terminology (e.g., use of “Half IRONMAN®” versus “IRONMAN® 70.3”), should be addressed to improve clarity, but these do not detract from the overall quality of the work.

In summary, this is a well-executed and informative systematic review that makes a meaningful contribution to the endurance sports literature. With minor revisions focused on clarity, consistency, and editorial refinement, the manuscript would be suitable for publication.

Answer: We agree with the expert reviewer and we thank reviewer 1 for the positive and constructive evaluation of our manuscript. We have revised the manuscript with:

Structured key elements in the discussion in a more concise and itemized manner to enhance readability.

Shortened long sentences where identified and appropriate.

Standardized terminology throughout the manuscript with consistent use of “IRONMAN® 70.3”.

Reviewer #2: Thank you for the opportunity to review the article entitled “Performance, Physiology, and Determinants of Success in IRONMAN 70.3: A Systematic Review”.

I find the article's objective to be of interest to the scientific community, as it is the first review paper on this specific triathlon distance. However, several important critical issues require significant effort on the part of the authors before publication can be considered. The main critical issues identified are: 1) lack of appropriate terminology for searching articles (the distance under consideration is commonly referred to as “middle-distance triathlon”, “70.3 IRONMAN” is the branded distance and therefore may only partially include articles published on the topic); 2) the “results” and “discussion” sections require extensive reworking, in particular, the results section contains too many sub-paragraphs presenting information in a non-systematic manner (different in terms of organisation within and between paragraphs) and often lacking information (results data, methodology), and often containing information not relevant to the subject of the study, while the discussion section is simply a discursive summary of the results section without the presence of numerical data and citations; 3) the structure of the tables needs to be re-evaluated to allow readers to see the information more clearly and correctly; 4) the “introduction” section already presents a summary of all results of the paper, leading the reader to wonder what is the novelty and usefulness of conducting this review.

I am also leaving a series of detailed comments, which are not exhaustive, in light of the critical issues that have emerged and been reported. I hope that my comments will help the authors to improve the quality of their work.

Answer: We agree with the expert reviewer and we thank reviewer 2 for the detailed and constructive feedback. The detailed comments were very helpful and contributed to improving the overall quality of our manuscript.

1) Search terminology: We tested the use of the term “middle distance triathlon”, but this approach resulted in a large number of irrelevant records. Therefore, we did not pursue it further. The term “IRONMAN 70.3” yielded the most relevant results for our research question. However, we repeated the search including the missing term and, by applying the hyphenated version “middle-distance triathlon”, we were able to identify two additional relevant studies, which have now been included.

2) Results and Discussion: We revised the results section by removing multiple subsections and restructuring it in a more systematic way. The studies are now consistently presented based on reported outcomes, sample size, methodology, and key results. In the discussion section, we added appropriate references and embedded the findings into a clearer scientific context.

3) Tables: We revised all tables and added key information including sex of participants, publication year, and authors to improve clarity and completeness.

4) Introduction: We revised the introduction and placed a stronger focus on the rationale and added value of this systematic review, reducing overlap with the results section.

Line 73 and 74 – the range of peak performance seems to be large…could you be more precise?

Answer: we agree with the expert reviewer and have narrowed down the numbers for the professional athletes.

Results: Participation has increased across age groups, with pronounced growth among female and masters’ athletes. Peak performance in professional male athletes is reached at age 28, and in female athletes at age 32.

Line 81 - What do you mean by “medical incidents”? Can you give some more information? And by ‘final race segment”, do you mean the running leg?

Answer: We agree with the expert reviewer and have clarified the meaning and the terminology.

Environmental heat and humidity impaired performance. Most medical incidents refers to acute events including hyperthermia, dehydration, hyponatremia, gastrointestinal distress, and musculoskeletal injuries occurred in the running leg.

Line 82 - In the results section of the abstract, you do not mention any information regarding the studies analysed in relation to the total number of participants, their specifications and the type of experimental design etc. It is therefore difficult to judge them. Please insert these information in the results section or modify this section.

Answer: We agree with the expert reviewer and have revised the abstract accordingly. We added information on the total number of participants, sample characteristics, and study design to improve the assessment of the included studies.

Line 116 – in fact, the mentioned distance is recognised as “middle-distance triathlon”, brended as 70.3 Ironman or half-Ironman by the Ironman company. Have you chosen to analyse only events organised by the Ironman brand? Why did you make this choice?

Answer: We agree with the expert reviewer and added “middle-distance triathlon”, at least we added 2 articles. The reason we excluded this search term was that the spelling without a hyphen generated a substantial number of irrelevant results.

Introduction section - All the information contained in this section, which should serve as a basic introduction to the topic addressed in the paper, has been presented in the results section. If the information gathered during this review is already known and established, what is the novelty and usefulness of conducting this review?

Answer: We thank the reviewer for this comment. The introduction was revised to focus solely on the general context and rationale for the review, removing any information that belongs exclusively to the results section. Regarding novelty: while some findings on IRONMAN® 70.3 exist in isolation, no prior systematic review has synthesized evidence across all relevant domains exclusively for this race distance. The present review therefore provides the first comprehensive, multidisciplinary synthesis specific to IRONMAN® 70.3, offering a structured evidence base that was previously unavailable to athletes, coaches, clinicians, and researchers.

Line 159 – May you provide further information about the “increasing scientific interest” in middle-distance tria

Attachment Submitted filename: PONE-D-25-68187_Revision 1_Answer to Reviewers.docx

# Peer review: Decision Letter 1

**DOI:** 10.1371/journal.pone.0352506.r003

**Authors:** Stefano Amatori

11 Jun 2026

Performance, Physiology, and Determinants of Success in IRONMAN® 70.3: A Systematic Review

PONE-D-25-68187R1

Dear Dr. Knechtle,

We’re pleased to inform you that your manuscript has been judged scientifically suitable for publication and will be formally accepted for publication once it meets all outstanding technical requirements.

Within one week, you’ll receive an e-mail detailing the required amendments. When these have been addressed, you’ll receive a formal acceptance letter and your manuscript will be scheduled for publication.

An invoice will be generated when your article is formally accepted. Please note, if your institution has a publishing partnership with PLOS and your article meets the relevant criteria, all or part of your publication costs will be covered. Please make sure your user information is up-to-date by logging into Editorial Manager at Editorial Manager® and clicking the ‘Update My Information' link at the top of the page. For questions related to billing, please contact billing support.

If your institution or institutions have a press office, please notify them about your upcoming paper to help maximize its impact. If they’ll be preparing press materials, please inform our press team as soon as possible -- no later than 48 hours after receiving the formal acceptance. Your manuscript will remain under strict press embargo until 2 pm Eastern Time on the date of publication. For more information, please contact onepress@plos.org.

Kind regards,

Stefano Amatori, Ph.D.

Academic Editor

PLOS One

Reviewers' comments:

Reviewer's Responses to Questions

**Comments to the Author**

1. If the authors have adequately addressed your comments raised in a previous round of review and you feel that this manuscript is now acceptable for publication, you may indicate that here to bypass the “Comments to the Author” section, enter your conflict of interest statement in the “Confidential to Editor” section, and submit your "Accept" recommendation.

Reviewer #1: All comments have been addressed

**********

2. Is the manuscript technically sound, and do the data support the conclusions?

The manuscript must describe a technically sound piece of scientific research with data that supports the conclusions. Experiments must have been conducted rigorously, with appropriate controls, replication, and sample sizes. The conclusions must be drawn appropriately based on the data presented.

Reviewer #1: Yes

**********

3. Has the statistical analysis been performed appropriately and rigorously?

Reviewer #1: Yes

**********

4. Have the authors made all data underlying the findings in their manuscript fully available?

The PLOS Data policy requires authors to make all data underlying the findings described in their manuscript fully available without restriction, with rare exception (please refer to the Data Availability Statement in the manuscript PDF file). The data should be provided as part of the manuscript or its supporting information, or deposited to a public repository. For example, in addition to summary statistics, the data points behind means, medians and variance measures should be available. If there are restrictions on publicly sharing data—e.g. participant privacy or use of data from a third party—those must be specified.

Reviewer #1: Yes

**********

5. Is the manuscript presented in an intelligible fashion and written in standard English?

PLOS ONE does not copyedit accepted manuscripts, so the language in submitted articles must be clear, correct, and unambiguous. Any typographical or grammatical errors should be corrected at revision, so please note any specific errors here.

Reviewer #1: Yes

**********

6. Review Comments to the Author

Please use the space provided to explain your answers to the questions above. You may also include additional comments for the author, including concerns about dual publication, research ethics, or publication ethics. (Please upload your review as an attachment if it exceeds 20,000 characters)

Reviewer #1: The manuscript presents a comprehensive and well-structured systematic review addressing performance, physiological responses, nutritional strategies, environmental influences, and medical considerations specific to IRONMAN® 70.3 events. The topic is timely and relevant, and the authors successfully address an important gap in the literature, as no previous review appears to have focused exclusively on this race distance.

The revised version demonstrates substantial improvement following the previous round of review. The authors have adequately addressed the methodological, structural, and editorial concerns raised by the reviewers. In particular, the Results and Discussion sections have been reorganized more systematically, the terminology has been standardized throughout the manuscript, additional methodological details and quantitative information have been incorporated, and the tables have been substantially improved in clarity and completeness.

The systematic review methodology is appropriate and transparently reported, including adherence to PRISMA 2020 recommendations, clearly defined eligibility criteria, and the use of suitable risk-of-bias assessment tools according to study design. The decision not to conduct a meta-analysis is adequately justified due to the heterogeneity of the included studies.

The conclusions are supported by the evidence presented and appropriately acknowledge the current limitations of the literature, including small sample sizes, predominance of male cohorts, heterogeneous methodologies, and limited sex-specific analyses. The manuscript is generally well written in standard scientific English and is presented in an intelligible and logical fashion.

Overall, the manuscript has been significantly strengthened through revision and now represents a valuable contribution to the endurance sports and triathlon literature. I have no further major concerns and consider the manuscript suitable for publication.

**********

7. PLOS authors have the option to publish the peer review history of their article (what does this mean?). If published, this will include your full peer review and any attached files.

If you choose “no”, your identity will remain anonymous but your review may still be made public.

**Do you want your identity to be public for this peer review?**  For information about this choice, including consent withdrawal, please see our Privacy Policy.

Reviewer #1: **Yes:** Álvaro Miguel-Ortega

**********

# Editor report: Acceptance letter

**DOI:** 10.1371/journal.pone.0352506.r004

**Authors:** Stefano Amatori

PONE-D-25-68187R1

PLOS One

Dear Dr. Knechtle,

I'm pleased to inform you that your manuscript has been deemed suitable for publication in PLOS One. Congratulations! Your manuscript is now being handed over to our production team.

At this stage, our production department will prepare your paper for publication. This includes ensuring the following:

* All references, tables, and figures are properly cited

* All relevant supporting information is included in the manuscript submission,

* There are no issues that prevent the paper from being properly typeset

You will receive further instructions from the production team, including instructions on how to review your proof when it is ready. Please keep in mind that we are working through a large volume of accepted articles, so please give us a few days to review your paper and let you know the next and final steps.

Lastly, if your institution or institutions have a press office, please let them know about your upcoming paper now to help maximize its impact. If they'll be preparing press materials, please inform our press team within the next 48 hours. Your manuscript will remain under strict press embargo until 2 pm Eastern Time on the date of publication. For more information, please contact onepress@plos.org.

You will receive an invoice from PLOS for your publication fee after your manuscript has reached the completed accept phase. If you receive an email requesting payment before acceptance or for any other service, this may be a phishing scheme. Learn how to identify phishing emails and protect your accounts at https://explore.plos.org/phishing.

If we can help with anything else, please email us at customercare@plos.org.

Thank you for submitting your work to PLOS ONE and supporting open access.

Kind regards,

PLOS ONE Editorial Office Staff

on behalf of

Prof. Stefano Amatori

Academic Editor

PLOS One
